# DeepHEMNMA: ResNet-based hybrid analysis of continuous conformational heterogeneity in cryo-EM single particle images

**DOI:** 10.3389/fmolb.2022.965645

**Published:** 2022-09-08

**Authors:** Ilyes Hamitouche, Slavica Jonic

**Affiliations:** IMPMC - UMR 7590 CNRS, Sorbonne Université, MNHN, Paris, France

**Keywords:** deep learning, cryo-EM single particle analysis, molecular flexibility, continuous conformational heterogeneity, normal mode analysis, HEMNMA, DeepHEMNMA

## Abstract

Single-particle cryo-electron microscopy (cryo-EM) is a technique for biomolecular structure reconstruction from vitrified samples containing many copies of a biomolecular complex (known as single particles) at random unknown 3D orientations and positions. Cryo-EM allows reconstructing multiple conformations of the complexes from images of the same sample, which usually requires many rounds of 2D and 3D classifications to disentangle and interpret the combined conformational, orientational, and translational heterogeneity. The elucidation of different conformations is the key to understand molecular mechanisms behind the biological functions of the complexes and the key to novel drug discovery. Continuous conformational heterogeneity, due to gradual conformational transitions giving raise to many intermediate conformational states of the complexes, is both an obstacle for high-resolution 3D reconstruction of the conformational states and an opportunity to obtain information about multiple coexisting conformational states at once. HEMNMA method, specifically developed for analyzing continuous conformational heterogeneity in cryo-EM, determines the conformation, orientation, and position of the complex in each single particle image by image analysis using normal modes (the motion directions simulated for a given atomic structure or EM map), which in turn allows determining the full conformational space of the complex but at the price of high computational cost. In this article, we present a new method, referred to as DeepHEMNMA, which speeds up HEMNMA by combining it with a residual neural network (ResNet) based deep learning approach. The performance of DeepHEMNMA is shown using synthetic and experimental single particle images.

## 1 Introduction

Deep Mind’s AlphaFold2 predicts 3D structures of proteins from their 1D amino-acid sequences and produces 3D models of similar quality as those that can be obtained with experimental methods, but it is limited to prediction of small static structures (Jumper et al., 2021). Therefore, the structure and dynamics of challenging, large (multi-subunit) and flexible complexes is still studied by experimental methods, such as cryogenic electron microscopy (cryo-EM) single particle analysis (SPA).

Cryo-EM SPA can be used to collect data of different coexisting conformational states of purified complexes. Different zones of the sample containing many copies of the same complex (referred to as particles) in random and unknown orientations are imaged in the cryogenic electron microscope without tilting the sample. Individual particle images are then extracted from the collected parallel electron-beam projection images. Advanced image processing algorithms and software are then needed to solve the heterogeneity of the particle orientations (three Euler angles), positions (shifts in *x* and *y* directions in the image plane), and conformations in the obtained set of single particle images, in order to calculate 3D reconstructions of the different coexisting conformational states (Jonić, 2017). During the data collection in the microscope, the low electron dose used to minimize the radiation damage of the sample yields highly noisy images, which complicates the task of disentangling the conformational, orientational, and translational heterogeneity.

Continuous conformational changes of biomolecular complexes (gradual transitions with uncountable intermediate conformational states) yield a particularly challenging type of heterogeneity for image processing algorithms (Jonić, 2017; Sorzano et al., 2019), in contrast to discrete conformational changes (e.g., two-state heterogeneity due to ligand binding and unbinding). The current cryo-EM SPA research is still mainly based on using biochemical procedures to make samples as conformationally homogeneous as possible and on using discrete-classification-based image processing methods, both of which facilitate obtaining 3D reconstructions at high resolution (Svidritskiy et al., 2014; Bai et al., 2015; Zhou et al., 2015; Abeyrathne et al., 2016; Banerjee et al., 2016; Hofmann et al., 2019; Nakane et al., 2020; Kato et al., 2021). These methods are better suited to discrete conformational changes and usually based on image classification into a beforehand arbitrarily chosen number of classes (Penczek et al., 2006; Scheres et al., 2007; Scheres, 2012; Lyumkis et al., 2013). They usually result in a small number of classes related to different conformational states, where similar classes are combined to yield 3D reconstructions of higher resolutions, whereas other classes are ignored, among which the classes related to no-particle images (“junk” particles).

However, the huge conformational heterogeneity due to continuous conformational changes of complexes should not be regarded only as an obstacle to high-resolution 3D reconstruction, but also as a unique opportunity to describe multiple coexisting conformations at once, even at lower resolutions. Indeed, unconstraining the flexibility of complexes biochemically and obtaining a low-dimensional representation of the full conformational space (containing all conformational states present in the sample) are prerequisites for getting information about the mechanisms of action of the complexes in health and disease, with or without different ligands involving continuous conformational transitions (Dashti et al., 2020).

The last decade was marked by an active research in methods to pave the way for a full exploration of larger degrees of continuous conformational heterogeneity (Dashti et al., 2014; Jin et al., 2014; Tagare et al., 2015; Haselbach et al., 2018; Dashti et al., 2020; Harastani et al., 2020; Lederman et al., 2020; Moscovich et al., 2020; Giraldo-Barreto et al., 2021; Punjani and Fleet, 2021). These methods aim at determining the full conformational distribution (also called conformational space, landscape, or manifold), based on which the images with similar conformations could be assembled in 3D reconstructions and, optionally, a displacement of a 3D model can be animated in this space without calculating 3D reconstructions (Jin et al., 2014; Harastani et al., 2020).

The problem of determining the particle conformation, orientation, and shift from images is an ill-posed inverse problem because the number of unknowns to be determined (the conformation, orientation, and shift for each particle image) is larger than the number of input data (the number of particle images), which combined with a low signal-to-noise ratio (SNR) of cryo-EM images makes the problem very challenging. The problem becomes well-posed by considering a low-dimensional representation of the conformational distribution, like a finite number of distinct conformations when assuming discrete conformational variability (Scheres, 2012; Lyumkis et al., 2013; Punjani et al., 2017)) or a small number of flexible motions when assuming continuous conformational variability (Dashti et al., 2014; Jin et al., 2014; Dashti et al., 2020; Harastani et al., 2020; Punjani and Fleet, 2021). Very recently, deep learning (DL) approaches started to attract attention regarding the problem of continuous conformational variability (Gupta et al., 2020; Zhong et al., 2021a; Chen and Ludtke, 2021; Rosenbaum et al., 2021). If DL models could be trained to interpret particles images in terms of the corresponding continuously changing conformations, orientations, and shifts (without the orientation and shift predetermination by conventional image alignment based on a finite number of distinct conformations), this would not only boost but revolutionize cryo-EM research, considering a tremendous speed of the inference using trained DL models.

Currently, to the best of our knowledge, only two journal publications of DL approaches for combined conformational, orientational, and shift heterogeneity include a validation with experimental cryo-EM images (CryoDRGN (Zhong et al., 2021a) and e2gmm (Chen and Ludtke, 2021)). These two DL approaches interpret the conformational heterogeneity in single particle images assuming known Euler angles and shifts of the particles. These rigid-body parameters are determined prior to DL, by classical cryo-EM classification and refinement methods. However, the angles and shifts obtained by discretizing the continuous conformational heterogeneity into a small number of average conformational states are likely inaccurate and the mentioned DL methods do not include any refinement schemes to refine these initial angles and shifts. The most recent version of CryoDRGN, CryoDRGN2 (Zhong et al., 2021b), makes use of a multi-scale exhaustive search of orientations and translations over a discretized 5D parameter space (by increasing the resolution of the search grid over multiple scales), which is a more efficient version of the parameter search than the branch and bound algorithm used in an earlier version of CryoDRGN known as CryoDRGN-BNB (Zhong et al., 2020). The orientation and translation determination in CryoDRGN2 is done prior to DL of the volume or interleaved with it. The alternating between the pose determination and the volume updates is expected to refine the poses, which in turn should improve the volume learning. However, as the neural network training objective changes during the course of training because of alternating between the pose search and the volume learning, the method suffers from the problem of vanishing gradients (Zhong et al., 2021b). A different group of DL methods consider conformational homogeneity and train the network to learn orientations and translations, in the context of obtaining a preliminary 3D model from cryo-EM images (Miolane et al., 2020; Levy et al., 2022).

In the context of continuous conformational heterogeneity, the angles, shifts, and conformations should ideally be determined simultaneously and refined iteratively (Jonić, 2017), which is the case of our previously developed method, HEMNMA (Jin et al., 2014; Harastani et al., 2020). In HEMNMA, image analysis is integrated with the analysis of the motion directions simulated by the so-called normal mode analysis (NMA) (Tirion, 1996; Tama and Sanejouand, 2001; Suhre and Sanejouand, 2004; Skjaerven et al., 2009; Bahar et al., 2010; Nogales-Cadenas et al., 2013; Jonić and Sorzano, 2016; López-Blanco and Chacón, 2016) of a given atomic structure or a given 3D EM map (if no atomic-coordinate structure of the complex is available but a 3D reconstruction from cryo-EM images can be obtained), through the determination of the motion amplitudes along these directions for each single particle image independently of other images. The provided atomic structure or EM map facilitate the simultaneous determination of the particle angles, shifts, and conformations from noisy cryo-EM images by HEMNMA. The atomic structure or EM map used to obtain the normal modes (the simulated motion directions) is often referred to as the reference. It represents an initial conformation that is iteratively elastically deformed, oriented, and shifted for each particle image, until it matches the conformation, orientation, and shift of the particle in this image. The parameters of the conformational model, determined by HEMNMA for each particle image, are the amplitudes of the normal modes. The conformational parameters obtained by HEMNMA for all particle images are then shown in a common low-dimensional space, which allows 3D reconstructions from interactively grouped images with similar particle conformations and animated displacements of the reference in this space without 3D reconstruction.

However, HEMNMA is highly computationally demanding. The use of larger sets of normal modes and particle images requires longer processing times. To speed up HEMNMA data processing, we have developed a method that combines HEMNMA with supervised regression-task DL based on a convolutional neural network (CNN). The new method is referred to as DeepHEMNMA and is based on ResNet 34 CNN (He et al., 2016). In DeepHEMNMA, the network is trained to learn the relationships between a small set of particle images and the corresponding conformational and pose parameters of the particles (normal-mode amplitudes, orientations, and shifts) calculated by analyzing these images with HEMNMA prior to network training. Then, the trained network can be employed to predict (infer) the unknown conformational and pose parameters of the particles from a large set of particle images that were not used for the training.

In a short conference article (Hamitouche and Jonić, 2021), we presented a network (and showed its performance using synthetic data) to learn and predict the conformational parameters (normal-mode amplitudes), which allows animations of the reference in the obtained conformational space but not 3D reconstructions. In the meantime, we have extended this neural network approach to learn and predict the pose parameters as well (3 Euler angles and 2 shifts), which allows calculating 3D reconstructions in the predicted conformational space, using the predicted orientations and shifts for the groups of images with similar conformations interactively selected in this space.

In the present article, we describe DeepHEMNMA for learning and predicting all three types of the unknown parameters (conformational, orientational, and shift parameters) and show its performance using synthetic and experimental single particle cryo-EM data.

## 2 Methods

**FIGURE 1:**
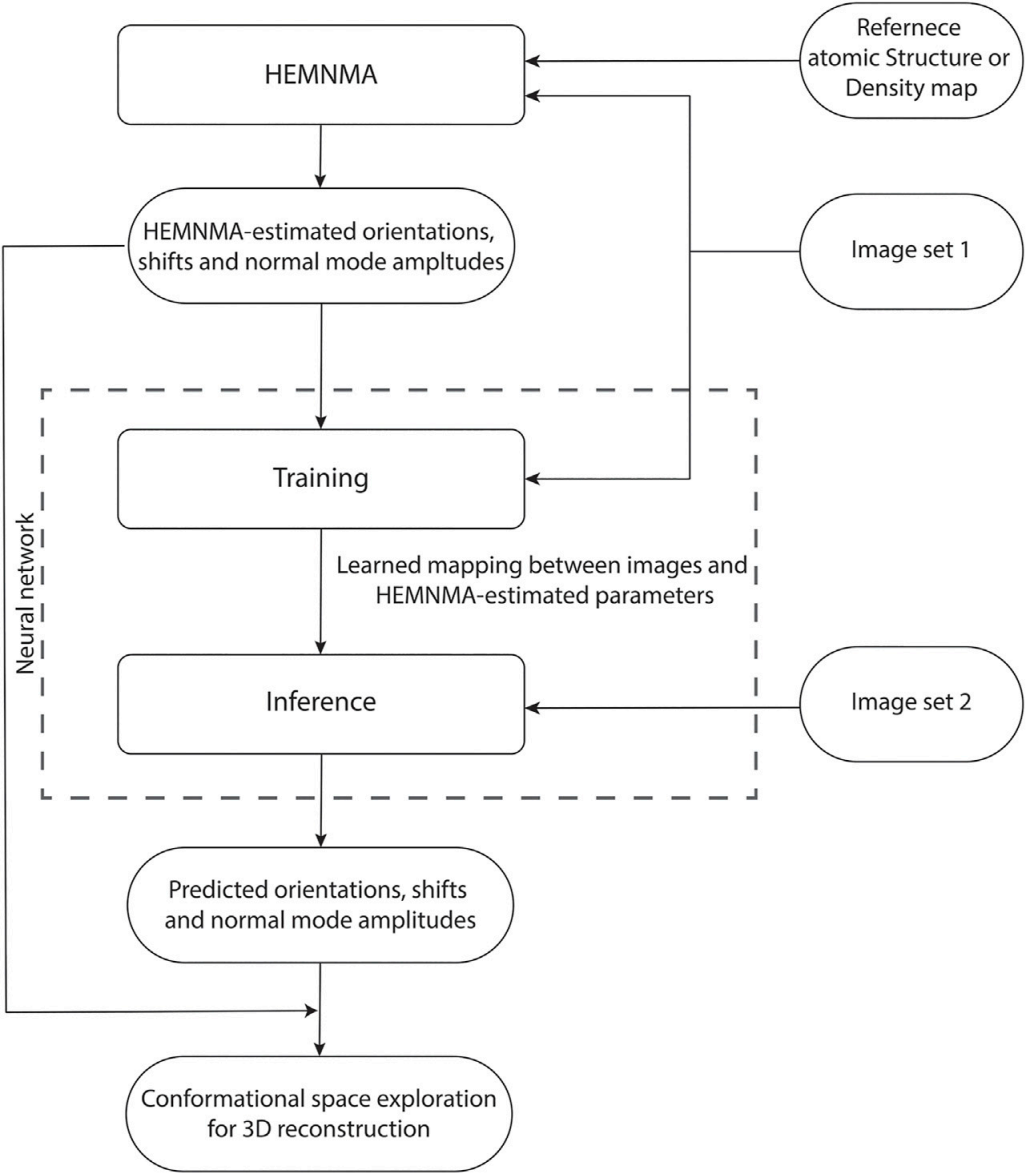
Flowchart of DeepHEMNMA combining HEMNMA and deep neural network methods. It uses an input atomic structure or EM map (reference) and an input set of images split into two subsets indicated as Image set 1 (referred to as training set) and Image set 2 (referred to as test set).

DeepHEMNMA workflow is shown in Figure 1 and has three stages. It uses an input set of images split into two subsets (indicated as Image set 1 and Image set 2 in Figure 1) and an input atomic structure or EM map (the reference for HEMNMA rigid-body and elastic alignment). In the first stage, HEMNMA is used to estimate the conformational (normal-mode amplitudes), orientational, and translational parameters for the images in Image set 1, through an iterative normal-mode-based elastic and rigid-body 3D-to-2D alignment of the reference with each single-particle image. In the second stage, the neural network is trained using Image set 1 (referred to as training set from now on) and the parameters estimated by HEMNMA for this set of images; then, the trained network is used to predict the parameters for the images in Image set 2 (referred to as test set from now on). The third stage consists of projecting the predicted conformational parameters onto a low-dimensional space and exploring this space, using a HEMNMA module. In this low-dimensional space, which could be considered as an essential conformational space, close points correspond to images with similar conformations and distant points to images with different conformations. The exploration of this space includes 1) generating animations of the displacement of the reference along the data distribution directions and 2) interactive grouping of images with similar conformations and calculating 3D reconstructions from these groups. In DeepHEMNMA, the parameters predicted by the network and those estimated by HEMNMA can optionally be combined into a single conformational space.

The deep neural network in DeepHEMNMA is a ResNet CNN feature extractor followed by a Multilayer Perceptron (MLP) block. The ResNet feature extractor consists of a ResNet 34 architecture (a 34-layer network) that extracts general relevant features from single-particle images. The MLP block predicts the conformational, orientational, and shift parameters based on the features extracted by ResNet.

In the remaining part of this section, we present the different steps of DeepHEMNMA in more detail.

### 2.1 Stage 1: HEMNMA estimation of the conformational and rigid-body parameters from the training images (Image set 1)

HEMNMA combines cryo-EM image analysis and NMA of the reference. It simultaneously estimates the conformational parameters (normal-mode amplitudes) and rigid-body parameters (orientations and translations) of the particle in each particle image. If the reference is an EM map, this EM map must be converted into a collection of 3D Gaussian functions, referred to as pseudoatoms (Jonić and Sorzano, 2016), before NMA can be performed.

**FIGURE 2:**
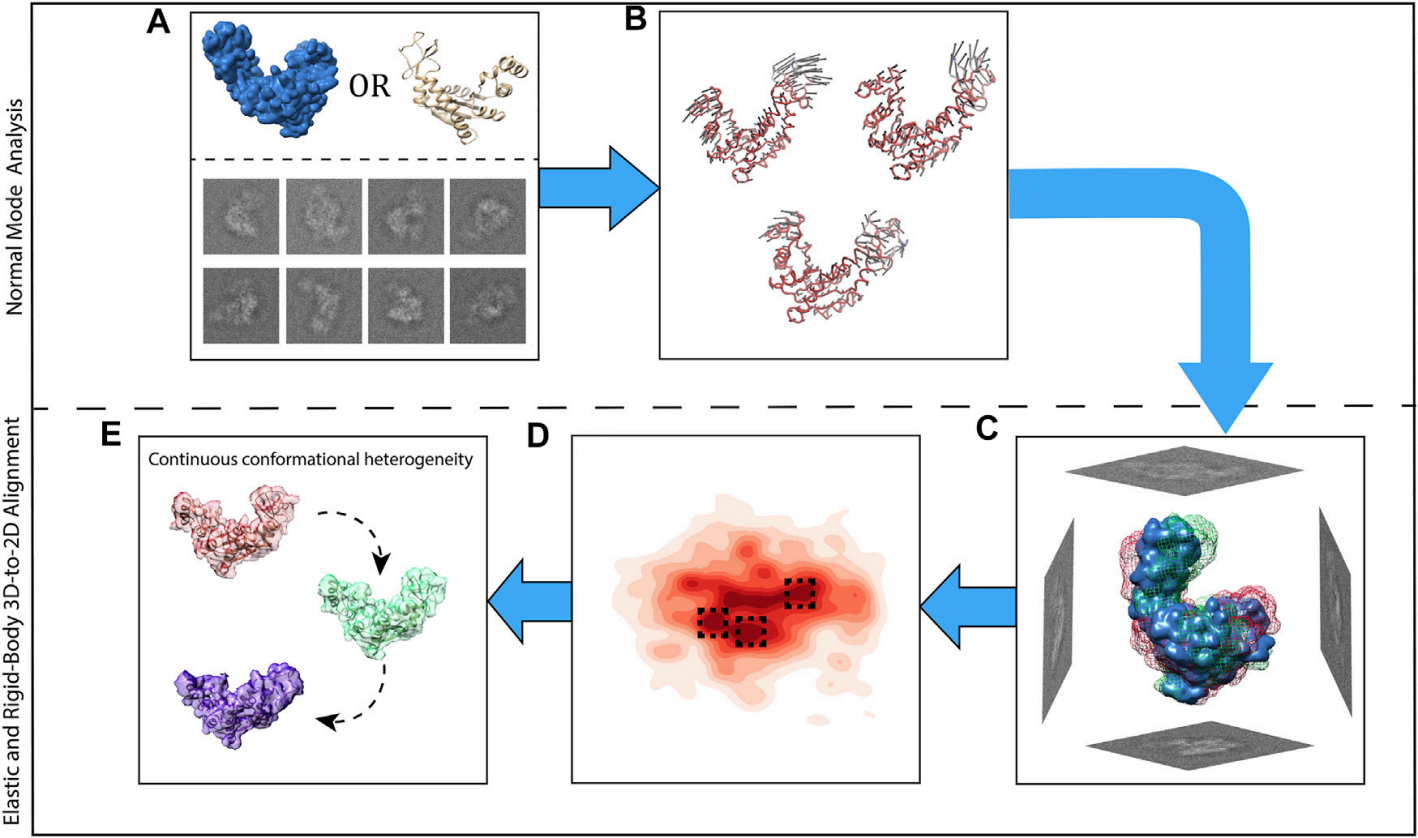
Graphical summary of HEMNMA steps. **(A)** Input EM map or atomic structure (the reference) and input single particle images. **(B)** Normal mode analysis of the reference and selection of normal modes (vectors). **(C)** Elastic and rigid-body alignment of each single particle image with the reference using the selected normal modes. **(D)** Mapping of single particle images onto a low-dimensional (here, 2D) conformational space in which the reference can be animated (denser regions are marked with a darker red color; close points correspond to images with similar conformations and distant points to images with different conformations). **(E)** 3D reconstructions from the densest areas in the low-dimensional conformational space shown by squares in **(D)**.

Figure 2 presents all steps of HEMNMA, which include NMA of the reference, iterative elastic and rigid-body 3D-to-2D alignment of the reference with particle images to estimate the conformational and rigid-body parameters of the particle in each image, projection of the estimated conformational parameters onto a low-dimensional conformational space, and analysis of the estimated conformational space in terms of animations of the reference and 3D reconstructions in the densest regions in this space.

We next briefly recall the theory of NMA and the iterative elastic and rigid-body 3D-to-2D alignment of HEMNMA, which are mandatory steps at Stage 1 of DeepHEMNMA. The projection of images onto a low-dimensional conformational space and the analysis of this space, which were originally developed for HEMNMA and are now also used in DeepHEMNMA, will be recalled at Stage 3 of DeepHEMNMA.

#### 2.1.1 Normal Mode Analysis

NMA is based on the so-called elastic network model (ENM) of the molecular system (Tirion, 1996), which is a simple and fast method to calculate vibrational modes and has been successfully used to predict biologically relevant motions (Ma, 2005; Tama et al., 2006; Skjaerven et al., 2009; Bahar et al., 2010; López-Blanco and Chacón, 2016). Normal modes are the vectors along which the system is displaced and are calculated using a harmonic approximation of the potential energy function of the system around a given, reference conformation. The reference conformation can be represented with atoms or with pseudoatoms (3D Gaussian functions with which an EM map, reconstructed from single particle images, can be represented (Jonić and Sorzano, 2016)). In the ENM, close atoms or pseudoatoms are connected with elastic springs (the interaction radius is a parameter that determines the size of the region beyond which the atom is not connected with other atoms and do not interact with them) (Tirion, 1996). Normal modes are calculated by diagonalizing the Hessian matrix (the matrix of the second derivatives of the potential energy function) (Tirion, 1996), which can be made faster in case of atomic structures by splitting the structure into blocks of consecutive residues (RTB blocks) that are only allowed to rotate and translate (Tama et al., 2000). Normal modes and their squared frequencies are eigenvectors and eigenvalues of the Hessian matrix, respectively. Lower-frequency normal modes describe more collective motions (displacing most of the atoms or pseudoatoms together, synergistically), whereas higher-frequency normal modes describe more localized movements of atoms. Several studies have shown that low-frequency normal modes correspond to functionally relevant biomolecular motions and that conformational transitions can be globally well described using a few low-frequency modes (Ma, 2005; Tama et al., 2006; Skjaerven et al., 2009; Bahar et al., 2010; López-Blanco and Chacón, 2016). Therefore, only a few low-frequency normal modes are usually selected for further analyses. The six lowest-frequency normal modes are not used, as related to rigid-body motions.

The elements of a normal-mode vector provide information on the direction of the displacement of each atom or pseudoatom with this normal mode (in HEMNMA, this displacement is in angstroms, Å, which are the standard atomic-coordinate units). The total number of normal modes and the length of each vector are equal to 3 times the number of atoms or pseudoatoms (the total number of the atomic or pseudoatomic coordinates). Atoms or pseudoatoms are displaced, to form a new conformation (model), using a linear combination of normal modes. Normal-mode amplitudes are the coefficients of the linear combination and indicate the contributions of the different normal modes to the global displacement (in HEMNMA, the normal-mode amplitudes have no physical units). NMA allows calculating normal modes (vectors of the displacement), but not the normal-mode amplitudes (amplitudes of the displacement along the vectors). The normal-mode amplitudes can be determined by fitting the conformational model with the experimental data, through numerical optimization of the coefficients of the linear combination of normal modes used for modeling, as described next.

#### 2.1.2 Iterative elastic and rigid-body 3D-to-2D alignment

In this step, HEMNMA iteratively maximizes a measure of similarity between a given particle image and the 2D projection of the reference conformation being elastically modified (using normal modes), rotated, and shifted, until the best elastic and rigid-body alignment is achieved between the image and the projection. It results in a quasi-simultaneous determination of the conformation (the coefficients of the linear combination of normal modes used for the conformational model, i.e., normal-mode amplitudes), orientation (three Euler angles), and position (two in-plane shifts) of the particle in each particle image. The HEMNMA-estimated parameters (normal-mode amplitudes, three Euler angles, and two in-plane shifts) are then used to train the neural network at Stage 2 of DeepHEMNMA.

### 2.2 Stage 2: Deep learning of the relationships between the training images and their HEMNMA-estimated parameters (Image set 1) and prediction of the unknown parameters from the test images (Image set 2)

At Stage 2, DeepHEMNMA uses a deep learning neural network, which accelerates the determination of the conformational and rigid-body parameters (normal-mode amplitudes, Euler angles, and in-plane shifts) for large sets of single particle images. This network is trained to learn the complex non-linear relationships between a subset of images (Image set 1) and their conformational and rigid-body parameters estimated at Stage 1 of DeepHEMNMA. The same network architecture is separately trained for each of the three types of parameters (normal-mode amplitudes, angles, and shifts). The three trained network models are then used to predict the three sets of parameters for the remaining subset of images (Image set 2 unseen by the network during the training).

**FIGURE 3:**
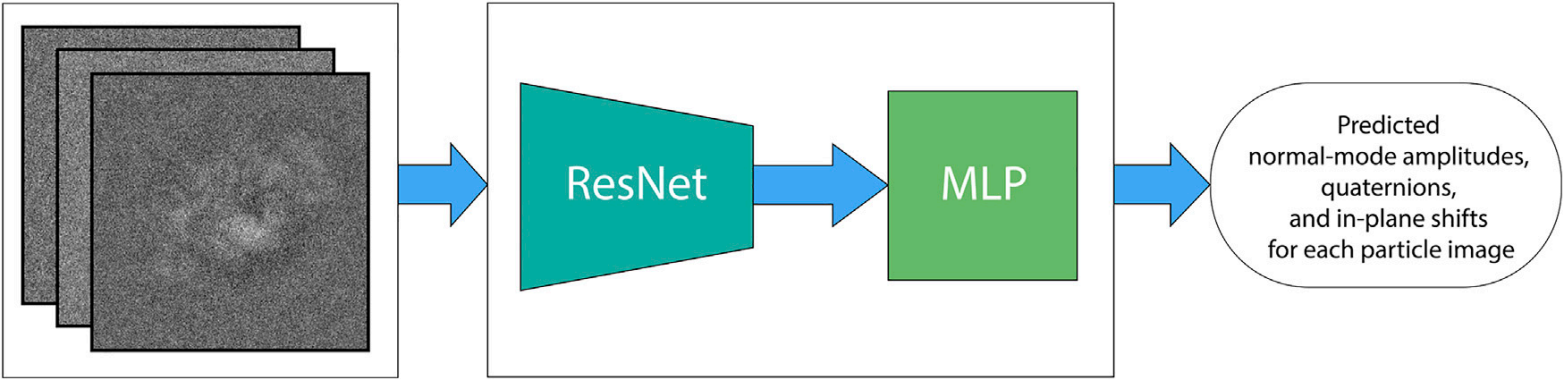
DeepHEMNMA neural network step. The deep learning neural network is a combination of a ResNet 34 feature extractor (ResNet block) and a 4-layer multilayer perceptron (MLP block). It is trained to map each single-particle image onto the corresponding, HEMNMA-estimated conformational parameters (M normal-mode amplitudes), orientational parameters (3 Euler angles), and positional parameters (2 in-plane shifts) of the particle in the image. DeepHEMNMA converts the Euler-angle representation of the orientation used in HEMNMA into a 4-parameter quaternion representation, which is learned by the neural network internally. The learned quaternion representation of the orientation is then converted back to the Euler-angle representation for the analysis at Stage 3 of DeepHEMNMA.

The neural network in DeepHEMNMA is a combination of a ResNet feature extraction block and an MLP estimator (predictor) block (Figure 3). Residual networks allow training of very deep CNNs, by introducing residual blocks (skip connections) in the network architecture (He et al., 2016). They are very effective as feature extractors and have shown great results in classification tasks (Tegunov and Cramer, 2019; Rappez et al., 2020). DeepHEMNMA uses ResNet 34 CNN architecture, which has 34 layers (He et al., 2016). In the training phase, ResNet 34 takes a subset of the input particle images (Image set 1) and extracts features that capture the pose (orientations and translations) and the motions of the biomolecular complex in the images. The extracted features are passed onto the MLP that maps them onto each of the three sets of parameters (normal-mode amplitudes, orientations, and translations). The training involves updating the weights of the whole network (ResNet and MLP blocks) to minimize the error of the parameter prediction by the network with respect to the parameters estimated by HEMNMA (mean absolute error type of loss), though Adam backpropagation stochastic optimization method (Kingma and Ba, 2014). The MLP takes the input flattened features maps, obtained by ResNet, and captures a multimodal distribution of the particle pose and motion parameters through a stack of 4 fully connected layers. The first 3 layers (1,000, 512, 128 nodes, respectively) have a nonlinear function (Rectified Linear Unit) applied to each layer, to model complex nonlinear functions. The last layer has the nodes with linear functions and their number is equal to the number of the output parameters. To prevent overfitting, one half of nodes in the MLP layers were randomly dropped out in each epoch and a weight decay of 10^−5^ (L2 regularization term (Krogh and Hertz, 1991)) was added to the gradients. In the test phase, the trained entire network model (ResNet and MLP blocks) predicts the pose and motion parameters of the particle from the remaining input particle images (Image set 2). The network is implemented using Python 3.8 and PyTorch 1.8.

DeepHEMNMA uses a unit quaternion representation for the orientation in 3D space, meaning that the three Euler angles estimated with HEMNMA for each single-particle image are converted into the corresponding quaternion and these quaternions are used to train the network. Similarly, the quaternions predicted by the network are converted back to the Euler-angle representation, for use with methods based on the orientation representation with Euler angles (the majority of cryo-EM methods), as the 3D reconstruction method used at Stage 3 of DeepHEMNMA. Quaternions provide an extensive representation of the orientations through a four-tuple system and help overcome the gimbal lock drawback of the representation by Euler angles (Hu et al., 2020). A basic information on the quaternion system and the conversion from Euler angles to quaternions and vice versa is provided in Supplementary Material SA. For more information, the reader is referred to the recent review (Hu et al., 2020). We have compared the performance of our deep learning network using the two representations and found that the network achieves slightly worse results with the Euler-angle representation (results provided in Supplementary Material SB). Therefore, we decided to use the quaternion representation for our deep learning network.

As the network is trained separately for each of the three types of parameters, the number of outputs in the final MLP layer is different for the three trained models (*M* outputs for *M* normal-mode amplitudes, 4 outputs for the quaternion representation of 3D orientation, and 2 outputs for the shifts in *x* and *y* directions in the image plane).

We have tested DeepHEMNMA with the ResNet architectures deeper than ResNet 34 (ResNet 50 and ResNet 101 having 50 and 101 layers, respectively) and found that the little improvement of the results obtained with such deeper networks does not to justify the extra time required for their training (the results provided in Supplementary Material SC).

In this article, the neural network training was performed on a 4-GPU computing node (NVIDIA V100, 5120 CUDA cores per GPU card) using a batch size of 2 and 400 epochs of Adam optimization method. The starting learning rate was $10^{-5}$ . The learning rate was gradually decreased by dividing it by 10 each 80 epochs.

The conformational parameters (*M* normal-mode amplitudes), orientational parameters (3 Euler angles obtained by conversion from 4-parameter quaternions), and translational parameters (2 shifts in *x* and *y* directions in the image plane) predicted at Stage 2 are then analyzed at Stage 3 of DeepHEMNMA, as explained next.

### 2.3 Stage 3: Conformational-space dimension reduction and analysis

At Stage 3 of DeepHEMNMA, a dimensionality reduction method is first used to project the set of *M* normal-mode amplitudes predicted by the neural network onto a lower-dimensional space (usually, a 2D or 3D space), which can then be visualized. The dimensionality reduction in DeepHEMNMA is a feature brought by HEMNMA. Several dimensionality reduction methods are available in HEMNMA and we usually use Principal Component Analysis (PCA), which is a widely used and intuitively clear dimensionality reduction method.

**FIGURE 4:**
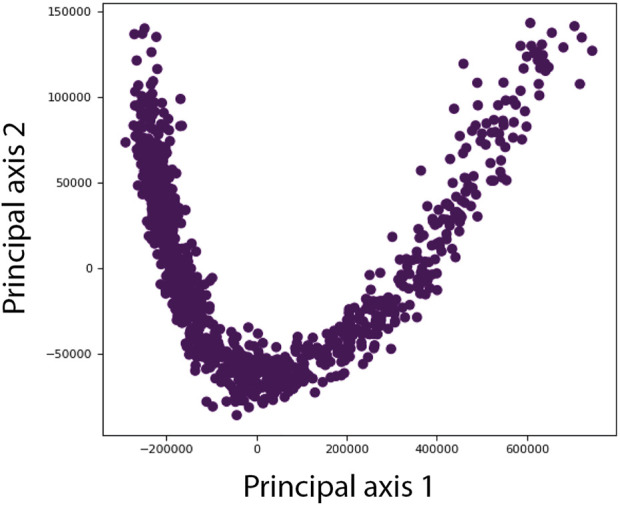
Illustration of a lower-dimensional (here, 2D) conformational space obtained by principal component analysis of the conformational parameters (normal-mode amplitudes) estimated by HEMNMA or predicted by the neural network of DeepHEMNMA. Different points represent different particle images. Each point corresponds to the conformational parameters (normal-mode amplitudes) of the molecular complex in the corresponding sigle-particle image. For each point, the orientation and position of the molecular complex are also available (estimated by HEMNMA or predicted by the neural network) and can be used to calculate 3D reconstructions from interactivelly selected groups of images with similar conformations in the densest regions of this space (not shown in this illustration but in the experiments below).

In the lower-dimensional conformational space (Figure 4), each point represents a conformation predicted for a given single-particle image and close points correspond to similar conformations. For each point, the predicted orientation and position of the particle in the image are also available and can be used to calculate 3D reconstructions from groups of images with similar conformations, interactively selected in high-density regions of this space (no automated clustering but a user’s choice of groups). The interactive grouping of images with similar conformations in DeepHEMNMA is also a feature brought by HEMNMA.

Beside using 3D reconstructions, the conformations predicted by the neural network can also be inspected by visualizing movies of the motion of the reference along the data distribution directions in this space. Additionally, it can be noted that the dimensionality reduction and further analysis can also be performed for the conformational space that combines the conformations estimated by HEMNMA and those predicted by the network.

## 3 Results

To evaluate the performance of DeepHEMNMA thoroughly, we carefully designed and run several experiments with synthetic datasets of the chain A of adenylate kinase (AK) from the PDB database (PDB:4AKE) (Müller et al., 1996) and with the experimental cryo-EM dataset of yeast 80S ribosome-tRNA complexes from the EMPIAR database (EMPIAR:10,016) (Svidritskiy et al., 2014). In this section, we describe these experiments and show their results.

### 3.1 Performance of DeepHEMNMA with synthetic data

In this section, we present results obtained with synthetic single particle images affected by noise and contrast transfer function (CTF) of the simulated microscope, to demonstrate the entire DeepHEMNMA protocol and show its accuracy and speed. The dataset was obtained by randomly sampling synthetic continuous conformational transitions, orientations, and positions of AK. The parameters of the synthetic AK conformation, orientation, and position were used as the ground-truth parameters to assess the accuracy of the prediction of these parameters by the neural network. As the network was trained using HEMNMA-estimated parameters, the accuracy of the neural network prediction was also assessed with respect to the HEMNMA-estimated parameters.

#### 3.1.1 Methods used to assess the neural-network prediction (inference) accuracy

The accuracy of the parameters predicted (inferred) by the neural network from images (normal-mode amplitudes, angles, and shifts) was assessed with respect to the ground-truth and HEMNMA-estimated parameters. The metrics to assess the accuracy of the inferred normal-mode amplitudes and shifts was the mean absolute error. The metrics to assess the accuracy of the inferred Euler angles was the average angular distance between the rotated coordinate-system axes (the inferred Euler angles mean the angles obtained by conversion from the inferred quaternions). As a complementary metrics to assess the accuracy of the inferred parameters, we used the root mean squared deviation (RMSD) between the atomic coordinates of AK displaced with the inferred and ground-truth parameters. More precisely, for each synthetic particle image, we calculated the RMSD between the AK atomic coordinates displaced with the inferred and ground-truth parameters using, for the displacement, one type of parameters at a time (normal-mode amplitude, angles, or shift). Then, we averaged the RMSDs over all images, for each parameter type separately. Additionally, we assessed the inference accuracy using 3D reconstructions from the groups of images with similar inferred conformations (the groups selected from different dense areas of the low-dimensional conformational space obtained by PCA of the inferred normal-mode amplitudes). We assessed the quality of each of these 3D reconstructions using Fourier Shell Correlation (FSC) with respect to the map simulated from the atomic model of conformation corresponding to the centroid of the image group used for 3D reconstruction.

#### 3.1.2 Data synthesis

**FIGURE 5:**
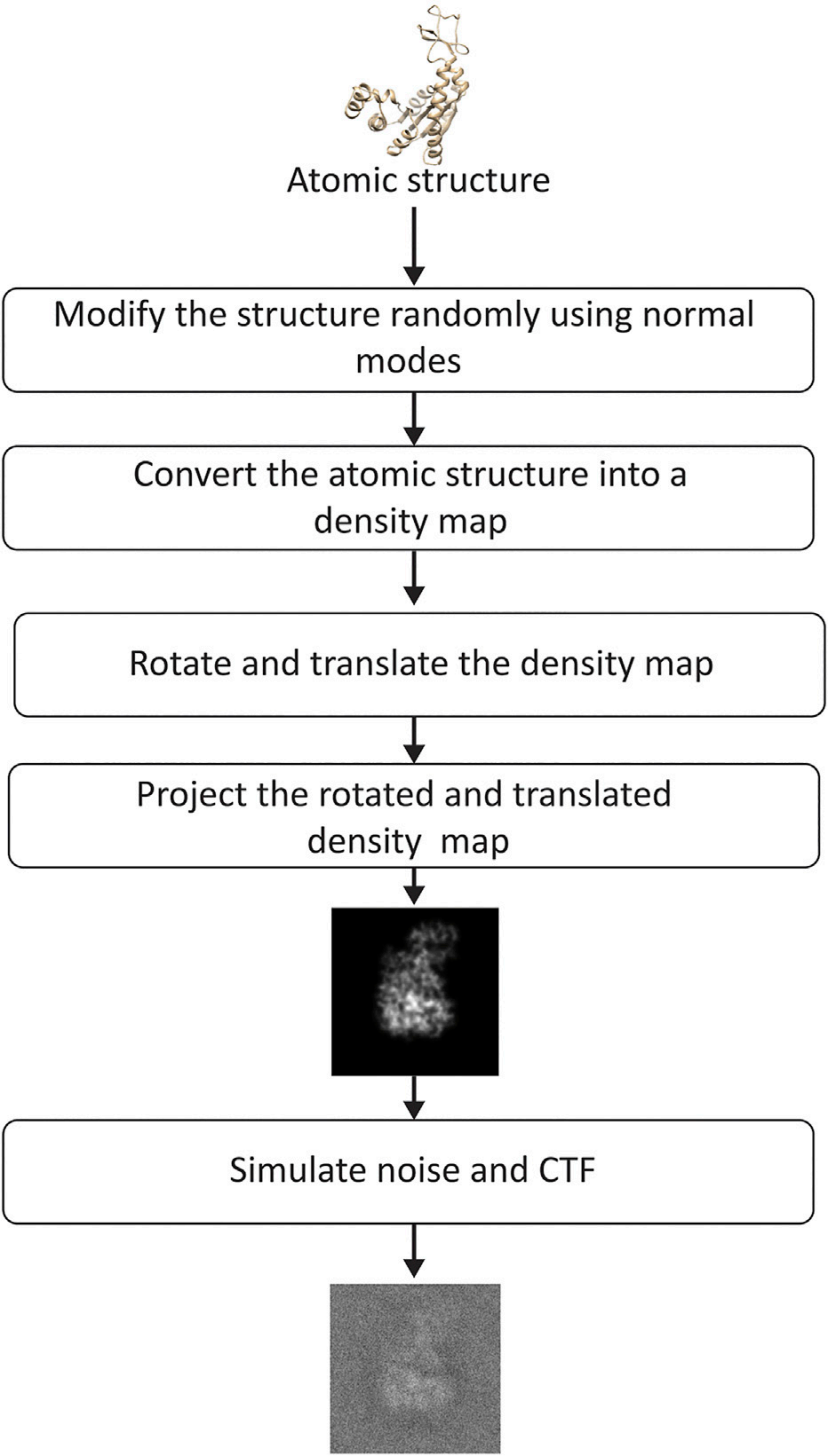
Flowchart of image synthesis for evaluating the performance of DeepHEMNMA. See the text for the details on the synthesis of random normal-mode amplitudes, angles, and shifts.

To synthesize the data for the experiment shown in this section, we followed the steps in the flowchart presented in Figure 5. The synthetic conformations were obtained by modifying the atomic AK structure using a linear combination of modes 7–9 (three lowest-frequency non-rigid normal modes), which is an arbitrary choice of normal modes made for this experiment. The linear combination of modes 7–9 was determined by their amplitudes $q_{7}$ - $q_{9}$ , respectively, which were randomly sampled from an arbitrary synthetic continuous conformational transition, as follows:

$$q_{7}\left(r\right)=-200\cdot r,\;q_{8}\left(r\right)=200\cdot \sin\left(\pi \cdot r\right),q_{9}\left(r\right)=200\cdot \cos\left(\pi \cdot r\right),$$ (1)

where r is a random variable, uniformly distributed between 0 and 1. It should be noted that the hypothetical ground-truth trajectory here, randomly sampled, has a helical shape that facilitates a qualitative (visual) inspection of the inference accuracy in the synthetic-data experiments shown in this article. Indeed, a quick visual inspection of the spread of the inferred points around the hypothetical ground-truth trajectory can be an additional indicator of the inference accuracy, beside the quantitative assessment by evaluating the parameter inference errors and 3D reconstructions.

The obtained conformations were then converted into density maps (Peng et al., 1996) (map size 256 × 256 × 256 voxels; voxel size: 0.325 Å × 0.325 Å × 0.325 Å). These maps were rotated and shifted using random angles and shifts (random uniform distribution) and, then, projected onto the image plane of size of 256 × 256 pixels (pixel size: 0.325 Å × 0.325 Å). The total number of synthesized images was 70, 000. It can be noted that the synthesized data are such that the conformation in each particle image can be unique (a different conformation can be present in each different image). The rotation followed the *ZYZ* angular convention, with the first and third rotation angles (around *z*-axis) between 0° and 360° and the second rotation angle (around *y*-axis) between 0° and 180°. The shifts were between -5 and +5 pixels in *x* and *y* directions. Finally, noise and CTF were applied to each synthesized image. Noise was applied before and after the CTF (a part of the noise was modulated by the CTF and the other not), as explained elsewhere (Sorzano et al., 2007). In the experiment shown in this section, the SNR was 0.1 and the CTF was simulated for a 200 kV microscope with a spherical aberration of 2 mm and a defocus of −0.5 µm. Additional experiments, regarding the influence of the number of images, noise, CTF, in-plane rotations, in-plane shifts, and image size, are shown in Supplementary Materials SD,E, and G.

#### 3.1.3 Experiment and results

The synthesized set of images was split into a training set of 20,000 images (Image set 1 in Figure 1) and a test set of 50,000 images (Image set 2 in Figure 1). Before running HEMNMA, the images were CTF-phase corrected (phase flip), as it would be done with experimental cryo-EM images. The CTF-phase flipped images were then downscaled to the size of 128 × 128 pixels (pixel size: 0.65 Å × 0.65 Å). The image size reduction was preceded by an antialiasing low-pass filtering, as usually done before image downscaling (in this case, the low-pass cutoff was 1.3 Å). Image size reduction not only speeds up processing, but also reduces noise in images, which generally yields better results, as also observed in our experiments (Supplementary Material SE compares the conformational prediction of the network trained using the original and downscaled images).

HEMNMA was used to estimate the normal-mode amplitudes, angles, and shifts for the training set of images (20,000 images). The images whose HEMNMA-estimated normal-mode amplitudes were far away from the majority were removed using the Mahalanobis distance measure (Mahalanobis, 1936). The Mahalanobis distance threshold of 3.2 was applied to the normal-mode amplitudes, which resulted in keeping 18,055 images for further processing. The network was trained using 14,055 images (from the kept 18,055 images). From the remaining 4,000 images, we used 2,000 images for tuning the network’s hyperparameters (the step referred to as validation in neural network terminology). The remaining 2,000 images were used for quickly testing and comparing the finally trained models and this set of images will here be referred to as small test set. The test set of 50,000 images was used to test the finally selected trained model and will here be referred to as large test set. In this section, we show the results of both tests (with 2,000 and 50,000 images).

**FIGURE 6:**
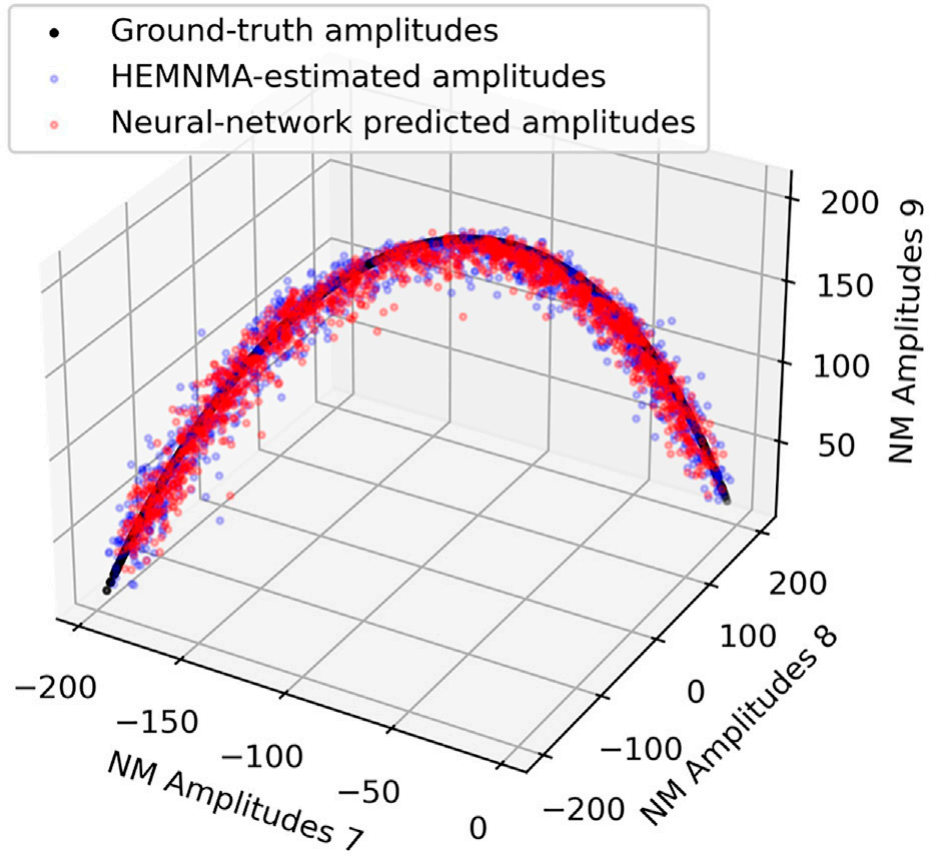
Overlap between inferred, ground-truth, and HEMNMA-estimated values of conformational parameters (normal-mode amplitudes) for a small test set of 2,000 synthetic images. Each point corresponds to an image and a molecular conformation inside it. Close points correspond to similar conformations and vice versa. For 2D scatter plots of the normal-mode amplitudes, see Supplementary Figure S2. See also Tables 1, 2.

Table 1 shows the distance (mean and standard deviation) of each inferred parameter with respect to its ground-truth and HEMNMA-estimated values, obtained using the small test set (2,000 images), and also includes the distance between the HEMNMA-estimated and ground-truth values for the same test set. The distance between the inferred and ground-truth values of each parameter, expressed in RMSD terms, is shown in Table 2. For the metrics used, please recall Section 3.1.1. An overlap between the inferred, ground-truth, and HEMNMA-estimated normal-mode amplitudes obtained using the small test set is provided in Figure 6, which shows that the inferred normal-mode amplitudes follow the ground-truth continuous conformational transition globally well. The distances between the inferred and ground-truth values of parameters obtained using the large test set (50,000 images) and these distances expressed in RMSD terms are shown in Tables 3, 4, respectively. These tables show the same range of the parameter inference errors for the small and large test datasets, which indicates that the network model has successfully generalized during the training.

**TABLE 1:** Mean and standard deviation (Std) of the distance between inferred, ground-truth, and HEMNMA-estimated values of parameters (normal-mode amplitudes, angles, and shifts) for a small test set of 2,000 synthetic images (the data used for quick tests at the training step).

Parameter distance	Normal-mode amplitudes							Angles [°]		Shifts X [Å]		Shifts Y [Å]	
	Mean over modes 7–9	Mode 7		Mode 8		Mode 9							
		Mean	Std	Mean	Std	Mean	Std	Mean	Std	Mean	Std	Mean	Std
Inferred vs. Ground-truth	7.5	5.4	6.5	8.2	9.2	8.9	10.5	2.5	3.3	0.2	0.1	0.2	0.1
Inferred vs. HEMNMA	6.9	5.4	6.7	7.3	9.0	7.9	9.6	1.9	3.4	0.2	0.1	0.2	0.1
HEMNMA vs. Ground-truth	6.6	5.7	8.4	6.2	7.2	7.8	7.2	1.0	0.9	0.2	0.2	0.2	0.2

**TABLE 2:** Mean and standard deviation (Std) of the distance between inferred and ground-truth parameters from Table 1 (for a small test set of 2,000 synthetic images), but expressed in RMSD terms.

RMSD	Normal-mode amplitudes [Å]		Angles [Å]		Shifts [Å]	
	Mean	Std	Mean	Std	Mean	Std
Inferred vs. Ground-truth	0.4	0.2	0.9	1.0	0.3	0.2

**TABLE 3:** Mean and standard deviation (Std) of the distance between inferred and ground-truth values of parameters (normal-mode amplitudes, angles, and shifts) for a large test set of 50,000 synthetic images (the data used to test the generalization of the finally trained network on a large set of images).

Parameter distance	Normal-mode amplitudes							Angles [°]		Shifts X [Å]		Shifts Y [Å]	
	Mean over modes 7–9	Mode 7		Mode 8		Mode 9							
		Mean	Std	Mean	Std	Mean	Std	Mean	Std	Mean	Std	Mean	Std
Inferred vs. Ground-truth	7.8	6.6	8.7	9.5	10.6	7.3	9.9	2.6	3.4	0.2	0.2	0.2	0.2

**TABLE 4:** Mean and standard deviation (Std) of the distance between inferred and ground-truth parameters from Table 3 (for a large test set of 50,000 synthetic images), but expressed in RMSD terms.

RMSD	Normal-mode amplitudes [Å]		Angles [Å]		Shifts [Å]	
	Mean	Std	Mean	Std	Mean	Std
Inferred vs. Ground-truth	0.4	0.2	0.9	1.2	0.3	0.2

**FIGURE 7:**
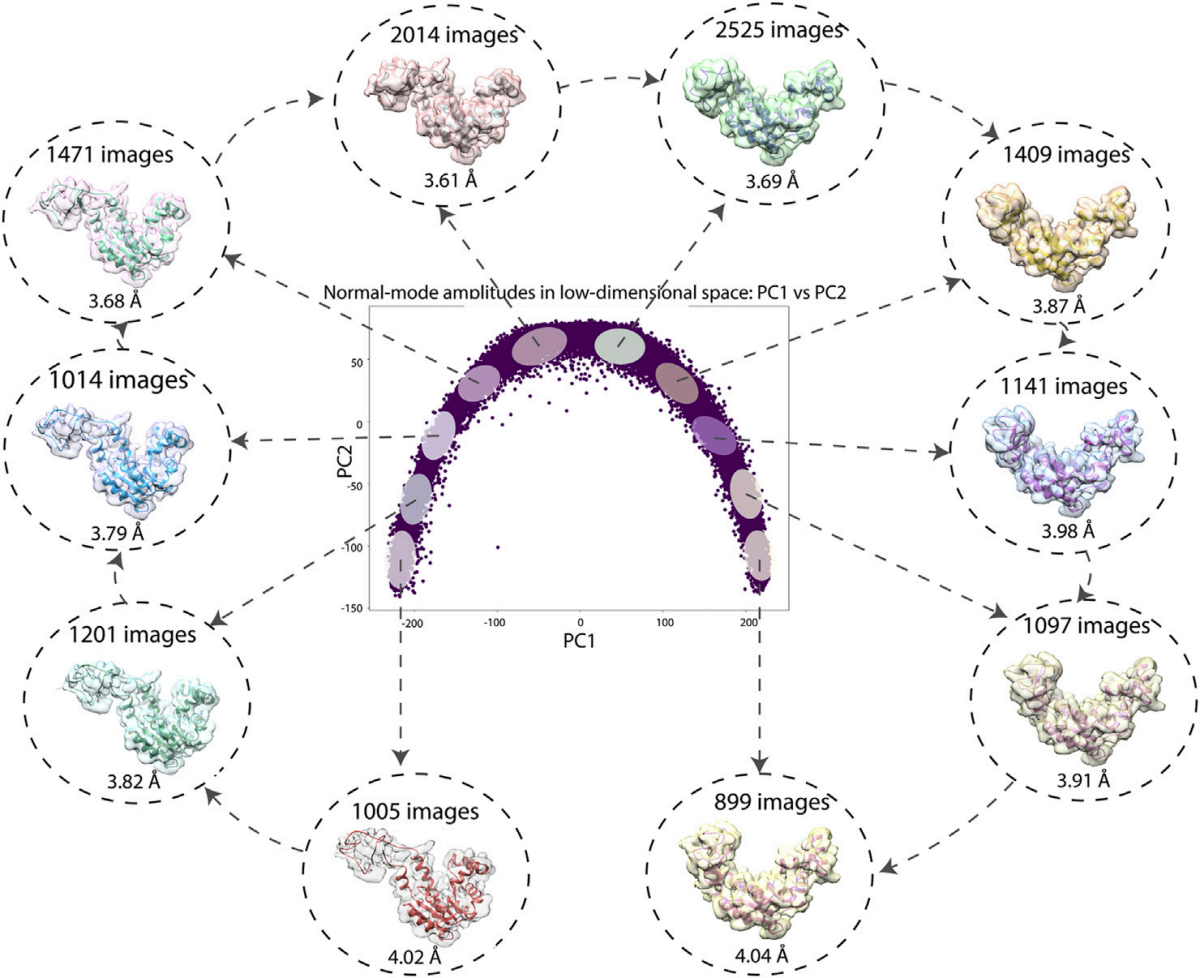
Low-dimensional (here, 2D) conformational space obtained by principal component analysis of the inferred conformational parameters (normal-mode amplitudes) for a large test set of 50,000 synthetic images, together with ten 3D reconstructions from ten different dense regions of this space supperposed with the corresponding atomic models (centroids of the regions). The network training and inference of normal-mode amplitudes, angles, shifts were done using images of size 128 × 128 pixels (for the inferrence accuracy, see Tables 3, 4) and the reconstructions were obtained from images of size 256 × 256 pixels. The number of images used for each reconstruction and the 0.5-FSC resolution of the reconstructed map are also shown (the FSC curves are provided in Supplementary Material SF). Each point in the conformational space corresponds to an image and a molecular conformation inside it. Close points correspond to similar conformations and vice versa.

Figure 7 shows a 2D conformational space obtained by PCA of the inferred normal-mode amplitudes. In this space, it is possible to calculate 3D reconstructions either from the reduced-size images (128 × 128 pixels), which were used for training and inference, or from the original-size images (256 × 256 pixels). Here, we demonstrate the reconstructions from the original-size images (using the inferred shifts, after their multiplication by 2, and the inferred angles). Ten 3D reconstructions were calculated from the images in the corresponding ten dense regions of the 2D PCA space. In Figure 7, each reconstructed map is overlapped with the atomic model that corresponds to the centroid of the region used for the reconstruction. Figure 7 also shows the number of images used for the reconstruction and the 0.5-FSC resolution of the reconstructed map with respect to the map simulated from the corresponding centroid atomic model. The resolution is in the range 3–4 Å (the average resolution according to the 0.143 and 0.5 FSC thresholds is 3.2 Å and 3.8 Å, respectively; for FSC curves, see Supplementary Material SF).

We have additionally calculated the resolutions of the maps reconstructed from the same-size subgroups of the ten groups of images (899 images in each subgroup, which is the number of images in the smallest of the ten groups), using the inferred, ground-truth, and HEMNMA-estimated angles and shifts, where the resolution was calculated with respect to the map simulated from the corresponding ground-truth centroid atomic model (obtained using ground-truth normal-mode amplitudes). The 0.5-FSC resolution of the 10 reconstructed subgroup maps is in the range 3.7–4.4 Å for the inferred parameters, 3.6–3.7 Å for the ground-truth parameters, and 3.7–4.4 Å for HEMNMA-estimated parameters. The 0.143-FSC resolution of the 10 reconstructed subgroup maps is in the range 3.1–3.3 Å for the inferred parameters, 3.0–3.1 Å for the ground-truth parameters, and 3.0–3.3 Å for HEMNMA-estimated parameters.

#### 3.1.4 Speed assessment

DeepHEMNMA is faster than HEMNMA alone and it is even faster for larger datasets. The wall-clock times of HEMNMA, network training, and network inference are provided in Supplementary Material SG (Supplementary Tables S5–S7, respectively) for two image sizes (256 × 256 and 128 × 128 pixels) and 3 normal modes (the number of normal modes used in the experiment with synthetic data in this article). HEMNMA was run on 160 INTEL 2.6 GHz CPU cores. The neural network was run on 4 GPU cards at the training step and on 1 GPU card at the inference step (NVIDIA V100 with 5120 CUDA cores per card). The estimated total number of computing hours needed by DeepHEMNMA for obtaining normal-mode amplitudes, angles, and shifts for 1 million synthetic AK images of size 128 × 128 pixels with 3 normal modes is around 44 times smaller compared to HEMNMA. Indeed, HEMNMA alone would require 64,000 CPU hours, whereas DeepHEMNMA would require 1,232 CPU hours and 233 GPU hours (Supplementary Material SG).

### 3.2 Performance of DeepHEMNMA with experimental data

In this subsection, we show the results of DeepHEMNMA using cryo-EM data of yeast 80S ribosome-tRNA complexes available in EMPIAR database under the accession code EMPIAR-10016 (Svidritskiy et al., 2014).

#### 3.2.1 Dataset

The dataset consists of a stack of single particle images of size 360 × 360 pixels and pixel size of 1.05 Å (normalized so that the average of the image is zero and the standard deviation is 10) and 5 metadata files containing the orientation and translation parameters for 5 image classes obtained in (Svidritskiy et al., 2014) using FREALIGN (Lyumkis et al., 2013). Two of these metadata files, with the parameters of 23,726 and 22,369 images, were used in (Svidritskiy et al., 2014) to reconstruct two cryo-EM maps, accessible in EMDB database with the codes EMD-5976 (rotated conformation with 1 tRNA at resolution of 6.2 Å) and EMD-5977 (nonrotated conformation with 2 tRNA at resolution of 6.3 Å), respectively.

#### 3.2.2 Data preprocessing and data splitting for neural network

After inspecting all 5 classes obtained in (Svidritskiy et al., 2014) (quality and number of images in each class as well as 3D reconstruction reproduced for each class), we decided to run DeepHEMNMA only on images used for reconstructing EMD-5976 and EMD-5977 (46,095 images in total). The other 3 classes seemed less “clean” (many images seem to contain different objects than ribosomes) and the number of images in these classes was much smaller. Before running DeepHEMNMA, images were CTF-phase flipped and downscaled to the size of 128 × 128 pixels (pixel size: 2.95 Å). Our preliminary tests with this experimental cryo-EM dataset have shown large angular prediction errors (with respect to HEMNMA estimation) for the network trained using 20,000 images (recall that this is the number of images used to train the network with synthetic data). Therefore, we decided to split the set of 46,095 images as follows: 1) 32,000 images for training; 2) 2,000 images for validation (adjusting hyperparameters of the network); 3) 12,095 images for testing (large test set), out of which 2,000 images for quickly testing and comparing the trained models (small test set). Images from both FREALIGN classes were uniformly distributed in each of these image subsets.

#### 3.2.3 Reference model and normal mode analysis

The reference model used by HEMNMA to calculate normal modes and to analyze images with these normal modes was a coarse-grain model of the nonrotated conformation, which was made by keeping only Cα and P atoms from the atomic model available in the PDB database under the code PDB:3j78 (the atomic model derived from EMD-5977 map in (Svidritskiy et al., 2014)). The coarse-grain model had 17,082 atoms (Cα and P). Its normal modes were calculated using RTB block size of 20 residues and the interaction radius of 20 Å.

#### 3.2.4 Selection of normal modes for image analysis with HEMNMA

Regarding the selection of normal modes, an option was to only select the mode that describes the rotation between the large and small subunits of the ribosome, which is often informative enough to separate different ribosome states, as shown in our previous work (Jin et al., 2014). However, we decided to include more normal modes to demonstrate, using this experimental dataset, the performance of our deep neural network learning and prediction of a larger number of normal-mode amplitudes. Therefore, in this work, we selected normal modes by analyzing the motion field between the conformations obtained in (Svidritskiy et al., 2014) with FREALIGN. More precisely, we performed flexible fitting of the coarse-grain reference model (obtained from PDB:3j78) into EMD-5976 map, using 7 lowest-frequency non-rigid-body normal modes (modes 7–13), by employing our normal-mode-based 3D-to-3D flexible fitting approach of HEMNMA-3D (Harastani et al., 2021). The 7 obtained normal-mode amplitudes indicate that all 7 modes contribute to the motion between the two conformations. From this set of modes, we selected 4 modes with the highest contribution (modes 7–9 and 11), among which the mode describing the rotation between the ribosome subunits.

#### 3.2.5 DeepHEMNMA data analysis

HEMNMA was run to analyze images with the 4 selected normal modes, to obtain the conformations (normal-mode amplitudes), Euler angles, and shifts corresponding to these images, which were then used for the network training. The trained network was used to predict (infer) the normal-mode amplitudes, Euler angles, and shifts for the test images. The inferred normal-mode amplitudes were analyzed by PCA and 3D reconstructions were calculated from groups of images in this space using their inferred Euler angles and shifts.

**FIGURE 8:**
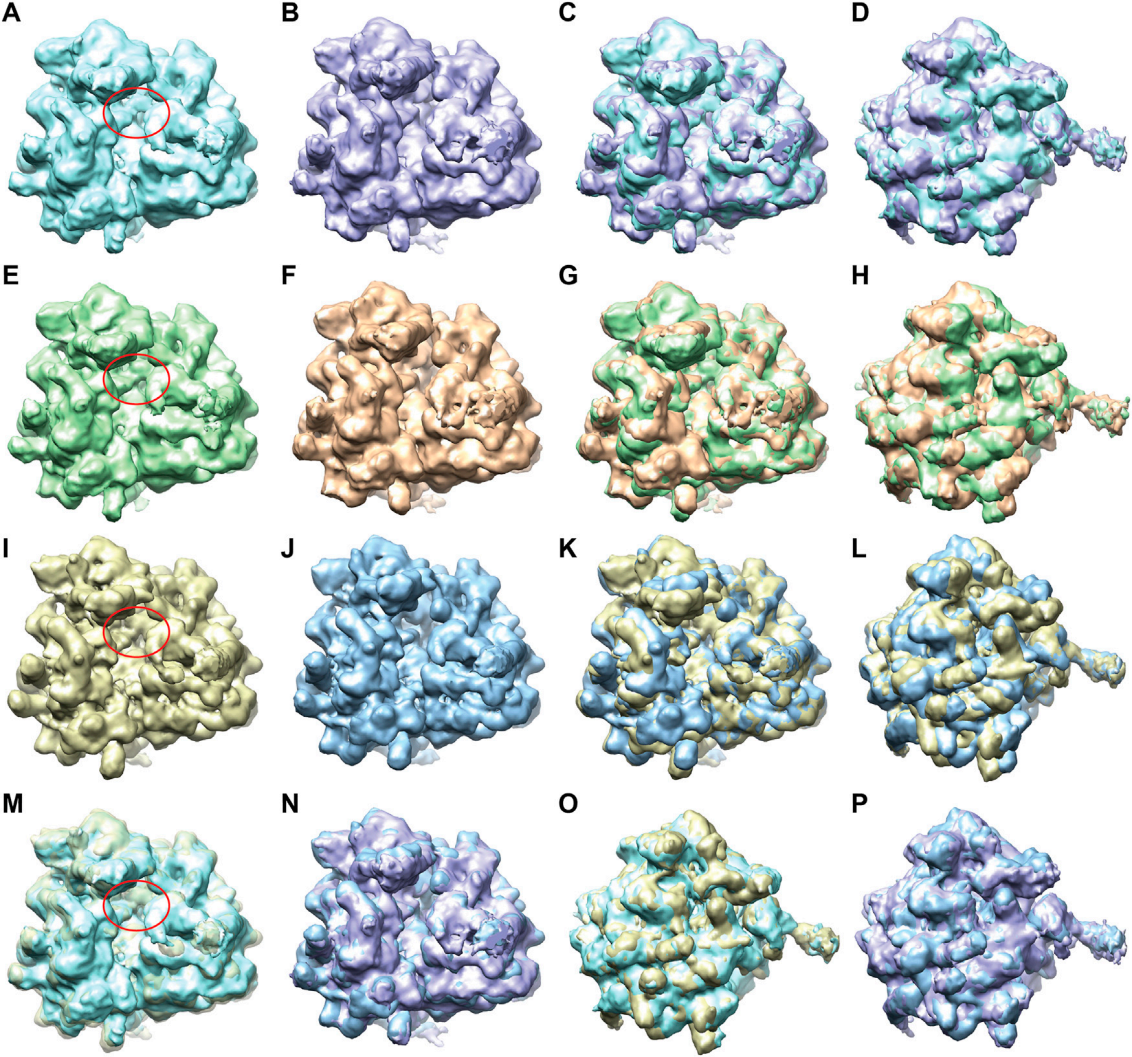
Two average conformations of yeast 80S ribosome-tRNA complexes obtained by 3D reconstruction from EMPIAR-10016 cryo-EM images, with and without additional mass in the region marked with a red ellipse, using DeepHEMNMA and using the original FREALIGN classification parameters from EMPIAR-10016 dataset. **(A,B)** Same view of two reconstructions obtained from the conformational space based on 12,095 images with inferred parameters (Supplementary Figure S4), from which groups of 4,741 and 4,219 images were used for the reconstructions. **(C,D)** Two views of the superposed reconstructions from A and B. **(E,F)** Same view of two reconstructions obtained from the conformational space based on 12,095 images with inferred parameters and 10,000 images with HEMNMA-estimated parameters (Supplementary Figure S5), from which groups of 7,870 and 6,682 images were used for the reconstructions. **(G,H)** Two views of the superposed reconstructions from E and F. **(I,J)** Same view of two reconstructions obtained using FREALIGN parameters for 22,369 and 23,726 images resulting in EMD-5977 and EMD-5976 maps, respectively. **(K,L)** Two views of the superposed reconstructions from I and J. (**M,N)** Superposition of the reconstructions obtained from images with inferred parameters and those obtained using FREALIGN parameters (M: overlap between the reconstructions shown in A and I; N: overlap between the reconstructions shown in B and J). **(O,P)** Different view of the superposed volumes shown in M-N, respectively. The red ellipse shown in panels A, E, I, M indicates the region with the additional mass (corresponding to the additional tRNA), with respect to the same region in panels B, F, J, N, respectively. All surfaces are shown in solid color except for the yellow surface in M that is shown transparent for a better visualization of the additional mass (red ellipse).

The 2D PCA space obtained for the set of 12,095 test images (Supplementary Figure S4) was split along the first principal axis into two groups of images, one with 4,741 images and the other with 4,219 images (Supplementary Material SH). The two 3D reconstructions obtained from these two groups (Figures 8A–D) indicate two different average conformations, with an additional mass in one reconstruction where the additional tRNA is expected (the region indicated by a red ellipse in Figure 8A) and without this additional mass in the other reconstruction (Figure 8B). The reconstructions obtained using FREALIGN metadata files from EMPIAR-10016 (Svidritskiy et al., 2014) (Figures 8I–L) show similarity with those obtained with DeepHEMNMA (Figures 8A–D and Figures 8M–P). Note however that the two reconstructions from FREALIGN metadata files were obtained using 22,369 and 23,726 images (related to EMD-5977 and EMD-5976 maps, respectively).

Furthermore, we found that the additional mass in the map reconstructed using inferred parameters (Figure 8A) could be better resolved if more images were used for this 3D reconstruction. We illustrate this by using a larger set of 22,095 images that was obtained by combining 1) 12,095 images with inferred parameters and 2) 10,000 images with HEMNMA-estimated parameters (from 32,000 images used for network training). The 2D PCA space for this set of 22,095 images (Supplementary Figure S5) was split along the first principal axis into two groups of images, one with 7,870 images and the other with 6,682 images (Supplementary Material SH). The 3D reconstructions from the latter two groups of images (Figures 8E–H) are similar to those obtained from the images with inferred parameters (Figures 8A–D) but some details are better resolved in Figures 8E–H, such as the additional mass related to tRNA (region marked by red in Figure 8E), which is directly linked to the use of more images for the reconstructions in Figures 8E–H.

**FIGURE 9:**
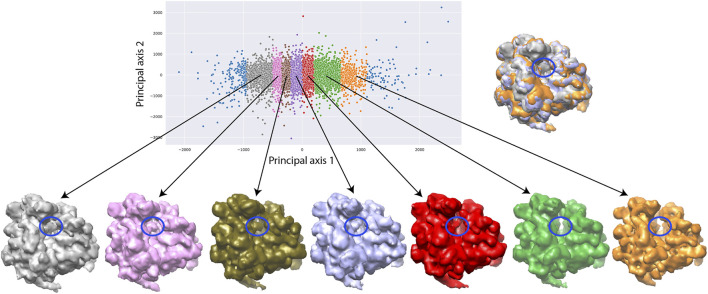
Two-dimensional conformational space of yeast 80S ribosome-tRNA complexes from the EMPIAR-10016 cryo-EM images, obtained by principal component analysis of normal-mode amplitudes inferred from 12,095 images (top, left), with 7 average conformations obtained by 3D reconstruction (bottom) from groups of images selected along the first principal axis, and an overlap of the maps reconstructed from the first (gray), fourth (violet), and seventh (orange) groups (top, right). The far left and far right images (blue) were not used for the reconstructions and the remaining part of the conformational space was split quasi-uniformly in the way to get at least 900 images per group. The reconstructions were obtained from 1,018, 1,148, 1,461, 1,816, 1,771, 975, and 949 images (bottom, from left to right). The color of the reconstructed map (bottom) corresponds to the color of the group of points in the conformational space (top). The blue ellipse overlapped with the reconstructed maps indicates the region where the additional mass, corresponding to the additional tRNA, is present or absent.

The need to use more images for 3D reconstruction in order to better resolve the tRNA could be explained by a larger conformational heterogeneity of the dataset. In Figure 9, we show more extensively the conformational variability using 3D reconstructions from a larger number of groups of images selected along the first principal axis of the 2D PCA space of the 12,095 images used for the inference. The PCA space was split quasi-uniformly in the way to get at least 900 images per group. One can note a variable degree of rotation between the small and large subunits as well as the presence and absence of the additional tRNA over the seven maps reconstructed from 1,018, 1,148, 1,461, 1816, 1771, 975, and 949 images (Figure 9).

This experiment shows that DeepHEMNMA can be useful for extensive analyses of conformational variability of biomolecular complexes, where large sets of experimental single particle images can be obtained. At least 30,000 particle images would be required for the neural network training. To take full advantage of the power of the trained network, one should aim at analyzing millions of single particle images. The analysis of such large datasets is not practical with conventional methods, whereas it has a low computational cost with trained neural networks.

## 4 Discussion and conclusion

This article introduces DeepHEMNMA, a hybrid method using HEMNMA image analysis (based on normal mode analysis) and a deep ResNet-based neural network to study continuous conformational variability of biomolecular complexes from single particle cryo-EM images. The purpose of the neural network is to accelerate HEMNMA-based continuous conformational landscape determination from cryo-EM images. DeepHEMNMA determines the conformational parameters (normal-mode amplitudes) and rigid-body parameters (three Euler angles and two in-plane shifts) of the biomolecular complex in each single particle images. To this goal, HEMNMA is first used to estimate these parameters from a subset of images. Then, the neural network is trained to learn the relationships between this subset of images and its HEMNMA-estimated parameters. The network is a ResNet 34 feature extractor followed by a multilayer layer perceptron. The trained network is then used to predict the parameters from the remaining images (unseen during the training). Finally, the conformational landscape is obtained by mapping the inferred normal-mode amplitudes onto a lower-dimensional space, which allows 3D reconstructions using the inferred angles and shifts. Also, this space allows animations of a model displacement and identification of possible hidden conformations.

We described this new approach and showed its performance with synthetic and experimental data. Using a synthetic dataset and a publicly available experimental dataset, we demonstrated a good generalization capability of the network (no overfitting against the training data), meaning that the trained network is able to accurately predict the conformation, orientation, and position of the molecule in the images that were not used for the training.

DeepHEMNMA has a general purpose and could be useful in analyzing conformational variability of various molecular complexes, as is the case for HEMNMA on which it is based. HEMNMA has been demonstrated on complexes of various sizes and architectures (Jin et al., 2014). It is thus expected that DeepHEMNMA performs like HEMNMA on the same complex. However, it should be noted that the network should be trained for each different molecular complex because each different complex will require a separate normal mode analysis, which depends on the shape of the complex.

We trained the network separately for normal-mode amplitudes, orientations, and shifts. This training strategy has the advantage that the number of images used for training can be adjusted for the different types of parameters. Indeed, with experimental data, we observed that learning of orientations requires around twice more images than learning of shifts or normal-mode amplitudes. However, in the future, we will add an option to our open-source DeepHEMNMA software to allow a combined training for all three types of parameters, which is expected to be faster than the separate training for each parameter type, for the same size of the training dataset.

DeepHEMNMA is a standalone method that can be used independently of other conformational variability methods (such as those of cryoSPARC, RELION, or Scipion) if a 3D model of one conformation of the complex can be provided (e.g., an atomic model from PDB database or a cryo-EM map from EMDB database). DeepHEMNMA does not use the particle pose information to learn the conformations (i.e., the normal-mode amplitudes, which together with normal modes describe the conformation). In DeepHEMNMA, the poses are only used for calculating 3D reconstructions from the groups of images selected based on similar conformations in the conformational space but not for obtaining this conformational space. The poses in DeepHEMNMA are obtained by prediction. Alternatively, after predicting the conformations, the poses could be determined by classical rigid-body 2D-to-3D alignment of the images with the density volumes simulated from the predicted conformations. This option could be useful in some cases, such as small number of images, and will be provided in the future. DeepHEMNMA was developed for analyzing large sets of images where pose prediction is more suitable. If the images are processed with other software before using DeepHEMNMA (e.g., cryoSPARC, RELION, Scipion, etc.), the poses determined by this software could be used instead, but their accuracy may be lower than the accuracy of the poses predicted by DeepHEMNMA or those determined by the rigid-body 2D-to-3D alignment of the images with the density volumes from DeepHEMNMA predicted conformations. This option may be provided in the future for the users willing to test it.

In this article, we reported the times required for the neural network training from scratch (without pre-training). Retraining a pre-trained model can help sometimes. However, when the datasets vary significantly in terms of conformational heterogeneity, SNR and CTF, and in particular with experimental data, we noticed that retraining a pre-trained model may take approximately the same time to converge as the training from scratch.

We tested different batch sizes for training (2, 8, 10, 16, 64, and 128) and obtained the best trade-off between processing time and accuracy with the batch size of 2. In particular, training with the batch size of 2 helped to avoid overfitting, together with other types of regularization (dropout and L2 regularization). Starting from $10^{-5}$ , the learning rate was divided by 10 each 80 epochs. After the third division, i.e., after epoch #240, we found that the network still learns while slowly stabilizing the kernels and MLP weights. The training and validation loss curves for the synthetic data experiment shown here are provided in Supplementary Material SI.

For running HEMNMA, the smallest recommended image size is 128 × 128 pixels. A reason for this is that HEMNMA uses a 2-level multiresolution data pyramid that includes further data downsampling (processing on the 64 × 64 pixel level first and then refining on the 128 × 128 pixel level). Another reason is that we recommend using HEMNMA with a rigid-body 3D-to-2D image alignment in wavelet domain (for robustness to noise) and the implemented wavelet transform requires the image size that is a power of 2. This was explained in the earlier publications of HEMNMA. We have not performed systematic tests of the neural network training with images smaller than 128 × 128 pixels.

As in the case of HEMNMA, DeepHEMNMA software is publicly available as part of ContinuousFlex (Harastani et al., 2020) plugin (https://github.com/scipion-em/scipion-em-continuousflex) for Scipion V3 (de la Rosa-Trevín et al., 2016), including graphical user interface giving the user the opportunity to easily use DeepHEMNMA on hybrid CPU-GPU architectures.

## Data Availability

The original contributions presented in the study are included in the article/Supplementary File. DeepHEMNMA software code is publicly available on Github (https://github.com/scipion-em/scipion-em-continuousflex) and is also part of the open-source ContinuousFlex plugin of Scipion V3. All questions regarding the software or data availability can be addressed to the corresponding author.
