# Supplementary material for: DeepHEMNMA: ResNet-based hybrid analysis of continuous conformational heterogeneity in cryo-EM single particle images
Source: Front Mol Biosci. 2022 Sep 8;9:965645. doi: 10.3389/fmolb.2022.965645 (PMC9493108; doi:10.3389/fmolb.2022.965645)
Supplement: Supplementary file 1 [file DataSheet1.pdf]

SUPPLEMENTARY MATERIAL TO:

DeepHEMNMA: ResNet-based hybrid analysis of continuous conformational heterogeneity in cryo-EM single particle images

Ilyes Hamitouche and Slavica Jonic

IMPMC - UMR 7590 CNRS, Sorbonne Université, MNHN, 4 place Jussieu, 75005 Paris, France

Corresponding author:
Slavica Jonic
IMPMC - UMR 7590 CNRS
Sorbonne Université, CC 115
4 place Jussieu, 75005 Paris, France
Phone : +33 1 44 27 72 05
Fax : +33 1 44 27 37 85
E-mail : slavica.jonic@upmc.fr

SA. Conversion between Euler angles and unit quaternions

A quaternion **q** is a 4-element vector that is defined as a hypercomplex number composed of a real part and three imaginary parts $\mathbf{q} = q_0 + q_1\mathbf{i} + q_3\,\mathbf{j} + q_4\mathbf{k}$, where the standard orthonormal basis for R^3 is given by three unit vectors **i** = (1, 0, 0), **j** = (0, 1, 0), and **k** = (0, 0, 1).

The Euler angle rotation that follows *ZYZ* convention (rotating about the *z*-axis first, then about the *y*-axis, and finally about the *z*-axis) can be converted into the following unit quaternion rotation:

$$q_{\phi\theta\psi} = q_\psi \otimes q_\theta \otimes q_\phi,$$

where

$$q_\phi = \begin{pmatrix} \cos\frac{\phi}{2} \\ 0 \\ 0 \\ \sin\frac{\phi}{2} \end{pmatrix}, q_\theta = \begin{pmatrix} \cos\frac{\theta}{2} \\ 0 \\ \sin\frac{\theta}{2} \\ 0 \end{pmatrix}, q_\psi = \begin{pmatrix} \cos\frac{\psi}{2} \\ 0 \\ 0 \\ \sin\frac{\psi}{2} \end{pmatrix},$$

leading to the following quaternion:

$$q_{\phi\theta\psi} = \begin{pmatrix} \cos\frac{\theta}{2}\cos\frac{\psi+\phi}{2} \\ -\sin\frac{\theta}{2}\sin\frac{\psi-\phi}{2} \\ \sin\frac{\theta}{2}\cos\frac{\psi-\phi}{2} \\ \cos\frac{\theta}{2}\sin\frac{\psi+\phi}{2} \end{pmatrix}.$$

Similarly, a 3 × 3 rotation matrix can be converted into the unit quaternion and the unit quaternion can be converted to a 3 × 3 rotation matrix, which makes the basis for converting quaternions back to Euler angles [49].

SB. Comparison of the use of Euler angles and quaternions for the neural network training

Supplementary Table S1 shows the accuracy of the angular inference for the network trained using Euler angles or using quaternions. The results shown for the network using quaternions are also shown in the main text (**Table 1**). It can be noted that the angular errors are larger when using Euler angles than when using quaternions.

Angular distance	Training with Euler angles [°]		Training with quaternions [°]	
	Mean	Std	Mean	Std
Inferred *vs.* Ground-truth	3.3	4.0	2.5	3.3
Inferred *vs.* HEMNMA	2.8	4.0	1.9	3.4
HEMNMA *vs.* Ground-truth	1.0	0.9	1.0	0.9

Supplementary Table S1 Mean and standard deviation (Std) of the distance between the inferred, ground-truth, and HEMNMA-estimated angles using a small test set of 2,000 images, after training with Euler angles or with quaternions using 14,055 images (image size: 128 × 128 pixels). The results for the use of quaternions are those shown in **Table 1**.

SC. Comparison of the network performance for different ResNet depths

Supplementary Table S2 compares the network performance for 3 different ResNet depths: 34 layers (ResNet 34), 50 layers (ResNet 50), and 101 layers (ResNet 101). This table shows that the best tradeoff between the speed and the accuracy is obtained using ResNet 34. Indeed, deeper feature extractors improve only slightly the results at the cost of much longer training times needed to train larger numbers of parameters.

ResNet depth (number of layers)	Distance between inferred and ground-truth normal-mode amplitudes							Approximate number of trainable network parameters (×10^6)	Training speed [hours]
	Mean over modes 7-9	Mode 7		Mode 8		Mode 9			
		Mean	Std	Mean	Std	Mean	Std		
34	7.5	5.4	6.5	8.2	9.2	8.9	10.5	24	19
50	7.3	5.1	6.3	8.1	9.0	8.8	10.2	26	22
101	7.2	5.0	6.2	8.0	8.9	8.7	10.1	47	42

Supplementary Table S2 Comparison of ResNets of 3 different depths (34, 50, and 101 layers) regarding the training speed, the number of the trainable network parameters, and the accuracy of the normal-mode amplitude inference (with respect to the ground-truth amplitudes), using a small test set of 2,000 images, after training with 14,055 images (image size: 128 × 128 pixels). The results of the use of ResNet 34 (the first row) are those shown in **Table 1**.

SD. Influence of number of images, noise, CTF, in-plane rotations, and in-plane shifts on conformational learning and prediction

Supplementary Table S3 shows results of tests of the network sensitivity to the number of images used for training, noise, CTF, and in-plane rotations and shifts, when training the network to learn the conformational parameters (normal-mode amplitudes). In these tests, we trained the network with ground-truth values of parameters, to evaluate the accuracy of the network independently of HEMNMA (instead of training the network with HEMNMA-estimated parameters, which is done in the main text).

The images used in the tests shown in this section were synthesized using a similar procedure to the one described in the main text. They had uniformly-distributed random projection directions (as described in the main text). The in-plane rotations and shifts were zero in one case and uniformly randomly distributed in the other case (in the range described in

the main text). The noise and the CTF were not applied in one case and applied in the other case (as described in the main text, using SNR=0.1 and -0.5 μm defocus). For these tests, we used a set of 10,000 images (size 256 × 256 pixels) and the same set after data augmentation to 20,000 images. The data augmentation was performed using the standard machine learning approach of making image copies by randomly rotating and shifting images from the original set. Each image from the set of 10,000 images was in-plane rotated using a random angle and in-plane shifted using random shifts in the range [-7,7] pixels (note that this shift range is slightly larger than the shift range used to synthesize the original images). In both cases, without and with data augmentation, we used 2,000 images for validation and 2,000 images for inference. The training was performed using the remaining 6,000 images from the set without data augmentation or using the remaining 16,000 images from the set with data augmentation. The images were not downscaled for the tests performed in this section.

Supplementary Table S3 shows that the inference error is lower for the network trained with 16,000 images than for the network trained with 6,000 images. However, the decrease in the inference error was not enough significant with the network trained with 30,000 images, considering the large computational cost of the training (not shown here), and we decided to perform all other experiments with synthetic AK data using 20,000 images at most.

Similar results to those shown in **Supplementary Table S3** were obtained using images with the CTF defocus of -1 μm (and SNR=0.1) and slightly better results were obtained using images with SNR=0.3 (for both -0.5 μm and -1 μm defocus values). Examples of synthesized images with two SNR values and two defocus values are shown in **Supplementary Figure S1**, indicating that images with SNR=0.1 and the defocus of -0.5 μm have lower contrast and less CTF-induced oscillations near the particle edges, meaning that they hold higher-resolution structural information. In this article, we show results using images with SNR=0.1 and -0.5 μm defocus.

Number of images for training	In-plane rotation	In-plane Shift	Noise	CTF	Distance between inferred and ground-truth normal-mode amplitudes						
					Mean over modes 7-9	Mode 7		Mode 8		Mode 9	
						Mean	Std	Mean	Std	Mean	Std
6,000	No	No	No	No	2.3	1.5	2.1	3.1	4.5	2.3	2.9
6,000	No	No	Yes	Yes	5.8	3.6	4.9	7.3	10.7	6.5	9.1
16,000	No	No	Yes	Yes	4.3	2.7	3.5	5.3	7.9	5.0	6.9
6,000	No	Yes	No	No	4.8	3.0	4.5	6.1	10.1	5.3	8.6
6,000	No	Yes	Yes	Yes	7.9	4.9	6.7	9.8	14.7	9.1	13.3
6,000	Yes	No	No	No	16.9	10.3	15.4	19.5	29.6	20.7	33.4
6,000	Yes	No	Yes	Yes	19.6	12.0	17.6	22.3	31.9	24.5	39.0
6,000	Yes	Yes	No	No	23.5	14.7	21.1	24.4	34.0	31.4	49.1
16,000	Yes	Yes	No	No	11.3	7.0	13.7	12.3	23.8	14.7	32.5
6,000	Yes	Yes	Yes	Yes	27.6	17.1	23.6	29.1	39.4	36.5	54.5
16,000	Yes	Yes	Yes	Yes	15.3	9.5	16.6	16.7	27.8	19.8	38.7

Supplementary Table S3 Accuracy of normal-mode amplitudes inferred for 2,000 synthetic images (size: 256 × 256 pixels) with and without in-plane rotations, shifts, noise (SNR=0.1), and CTF (defocus -0.5 μm), after the network training with ground-truth normal-mode amplitudes (to evaluate the accuracy of the network independently of HEMNMA). The gray rows denote that the training dataset was obtained by data augmentation.

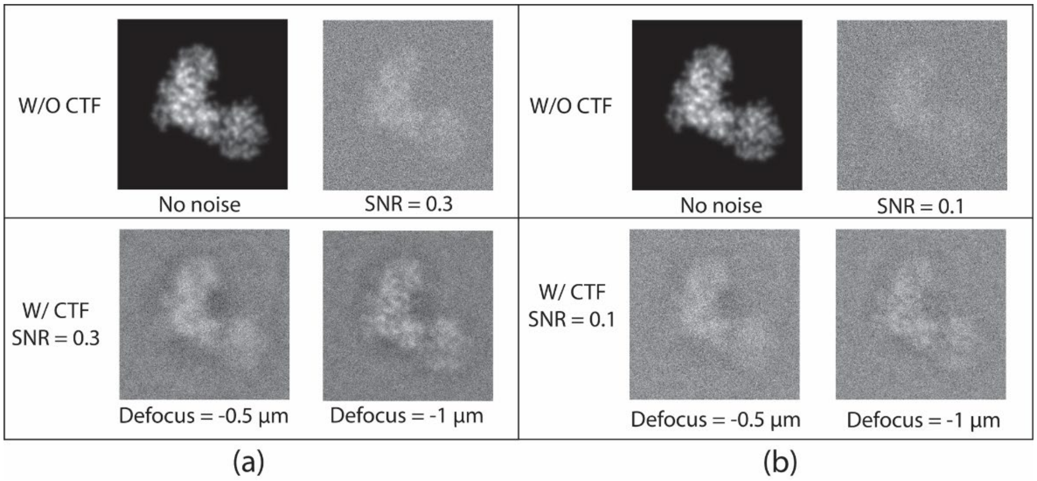

Supplementary Figure S1 Examples of noisy and CTF-affected images of Adenylate Kinase chain A (same view) synthesized with the SNR of 0.3 (a) and 0.1 (b) and with the CTF defocus of -0.5 µm (bottom left in (a) and (b)) and -1 µm (bottom right in (a) and (b)). Images without noise (top left in (a) and (b)) and without CTF (top right in (a) and (b)) are also shown.

SE. Influence of image size on conformational learning and prediction

Supplementary Table S4 and **Supplementary Figure S2** show accuracy of the inference of normal-mode amplitudes using the network trained with 14,055 synthetic images of 256 × 256 pixels and with these images downscaled to 128 × 128 pixels. The results obtained with the downscaled images are also shown in **Table 1** and **Figure 6** in the main text.

Image size	Distance between inferred and ground-truth normal-mode amplitudes						
	Mean over modes 7-9	Mode 7		Mode 8		Mode 9	
		Mean	Std	Mean	Std	Mean	Std
256 × 256 pixels	20.2	12.6	16.8	20.9	27.4	27.1	36.8
128 × 128 pixels	7.5	5.4	6.5	8.2	9.2	8.9	10.5

Supplementary Table S4 Influence of image size on the accuracy of conformational learning and inference. The inference was done using 2,000 synthetic images with the network trained with 14,055 images. The results for the size of 128 × 128 pixels (second row) are also shown in **Table 1**.

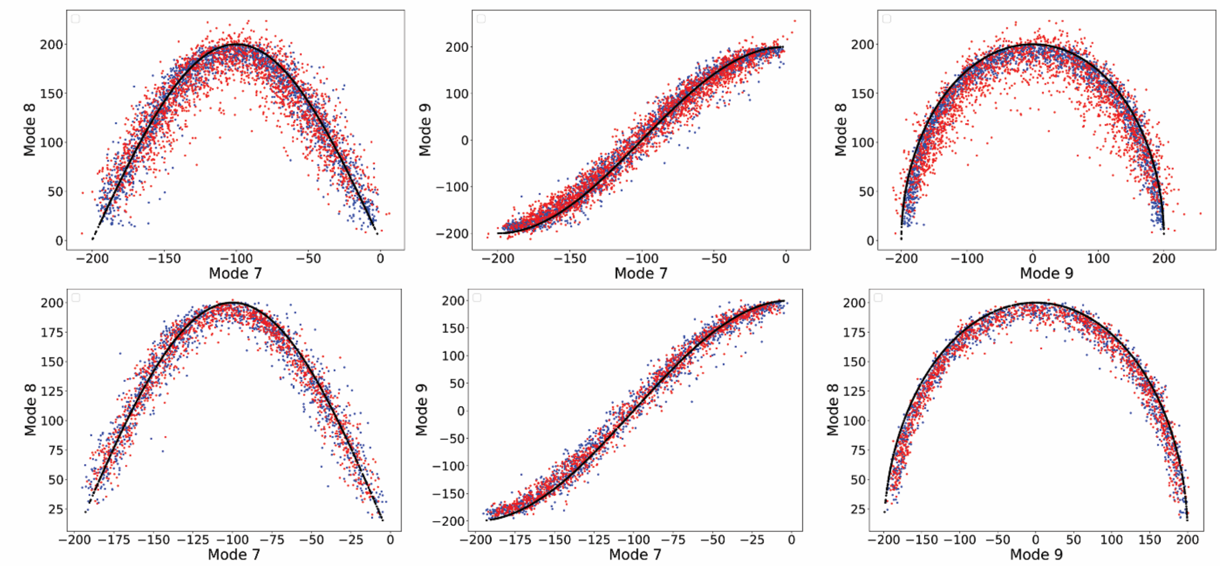

Supplementary Figure S2 Overlap between the inferred (red), ground-truth (black), and HEMNMA-estimated normal-mode amplitudes (blue) obtained using images of the size of 256 × 256 pixels (top row) and 128 × 128 pixels (bottom row). The results for the size of 128 × 128 pixels (bottom row) are also shown in **Figure 6** but as a 3D scatter plot. Each point corresponds to an image and a molecular conformation inside it. Close points correspond to similar conformations and vice versa. See also **Supplementary Table S4.**

SF. FSC curves of the reconstructions in the inferred conformational space from synthetic images

Supplementary Figure S3 shows FSC curves of ten 3D reconstructions from 10 regions of the conformational space shown in **Figure 7**. Each FSC was obtained with respect to the map simulated from the atomic model that is the centroid of the corersponding region used for the reconstruction. The reconstructed maps were neither filtered not masked before calculating the FSC curves. The maps and the number of images used for each reconstruction are shown in **Figure 7**. The intersections of the FSC curves with FSC=0.5 and FSC=0.143 indicate the map resolutions of 3.6-4 Å and 3.1-3.2 Å, respectivelly (**Supplementary Figure S3**).

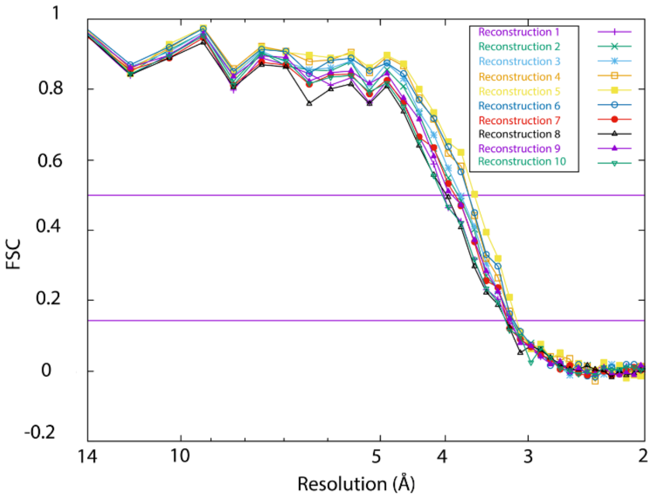

Supplementary Figure S3 FSC curves of ten 3D reconstructions from the corresponding ten regions of the conformational space shown in Figure 7, with respect to the maps simulated from the atomic-model centroids of the regions used for the reconstruction. The intersections of the FSC curves with FSC=0.5 and FSC=0.143 are also shown.

SG. Processing times of HEMNMA, network training, and network inference for synthetic images using three normal modes

Supplementary Tables S5-S7 show the wall-clock times needed for HEMNMA estimation, network training, and network inference using the synthetic data and 3 normal modes in the experiment shown in the main text. Note that the times in these tables are those of using one CPU core or one GPU card and should be multiplied by the number of CPU cores or GPU cards, respectively. Also, note that the time of HEMNMA is the time needed to estimate all parameters (normal-mode amplitudes, angles, and shifts), whereas the time of the network is the time needed for one type of parameters (normal-mode amplitudes, angles, or shifts) and should be multiplied by 3 for the 3 types of parameters.

**HEMNMA**	**1 image**	**20,000 images**	**10^6 images**
**256 × 256 pixels**	8 min	15.6 h	800 h
**128 × 128 pixels**	4 min	7.7 h	400 h

Supplementary Table S5 Wall-clock times needed for HEMNMA estimation of all parameters (normal-mode amplitudes, angles, and shifts). White and gray cells mean measured and estimated times, respectively. HEMNMA was run on 160 INTEL 2.6 GHz CPU cores. The indicated time (for one CPU core) should be multiplied by 160 to obtain the total number of computing hours.

**Training**	**6,000 images**	**14,000 images**	**50,000 images**
**256 × 256 pixels**	15 h	28 h	75 h
**128 × 128 pixels**	11 h	19 h	55 h

Supplementary Table S6 Wall-clock times needed for training the network to learn one type of parameters at a time (normal-mode amplitudes, angles, or shifts). White and gray cells mean measured and estimated times, respectively. The training was run on 4 NVIDIA V100 GPU cards. The indicated time (for using one GPU card) should be multiplied by 4 to get the total number of computing hours needed for one type of parameters, and the obtained time should be multiplied by 3 to get the total number of computing hours needed for all 3 types of parameters.

Inference	2 images	2,000 images	50,000 images	10^6 images
256 × 256 pixels	36 ms	0.3 min	7.5 min	2.5 h
128 × 128 pixels	6 ms	0.2 min	5 min	1.7 h

Supplementary Table S7 Wall-clock times needed for the trained network to infer one type of parameters at a time (normal-mode amplitudes, angles, or shifts). White and gray cells mean measured and estimated times, respectively. The inference was run on one NVIDIA V100 GPU card. The indicated time should be multiplied by 3 to get the total number of computing hours needed for all 3 types of parameters.

SH. Conformational space of experimental cryo-EM data of yeast 80S ribosome-tRNA complexes (EMPIAR-10016)

Supplementary Figure S4 shows the 2D conformational space obtained for the EMPIAR-10016 dataset, by PCA of the normal-mode amplitudes inferred from 12,095 images. It also shows two selected groups of images in this space, which were used for the 3D reconstructions shown in **Figure 8A** (4,741 images) and **Figure 8B** (4,219 images). The groups of images were selected automatically using logical operators on the coordinates of the two principal axes, which excludes some points that are far away from the majority and some points that are in the middle of the point cloud (the region with the coordinates [-100,100] on the principal axis 1 is excluded to get a clearer difference between the two 3D reconstructions from the selected groups of images). Such image grouping was done to demonstrate the reconstruction of two different average conformations of the ribosome from this space and to compare these reconstructions with those obtained based on the EMPIAR-10016 FREALIGN classification (**Figure 8**).

Supplementary Figure S5 shows the 2D conformational space obtained by PCA of a combined set of normal-mode amplitudes inferred from 12,095 images and normal-mode amplitudes estimated by HEMNMA from 10,000 images (the total number of images: 22,095 images). It also shows two selected groups of images in this space, which were used for the 3D reconstructions shown in **Figure 8E** (7,870 images) and **Figure 8F** (6,682 images). The merging of the inferred and HEMNMA-estimated normal-mode amplitudes was done to show the improvement of the 3D reconstructions with an increase in the number of images (in particular in the region where the additional tRNA is expected, **Figure 8E**).

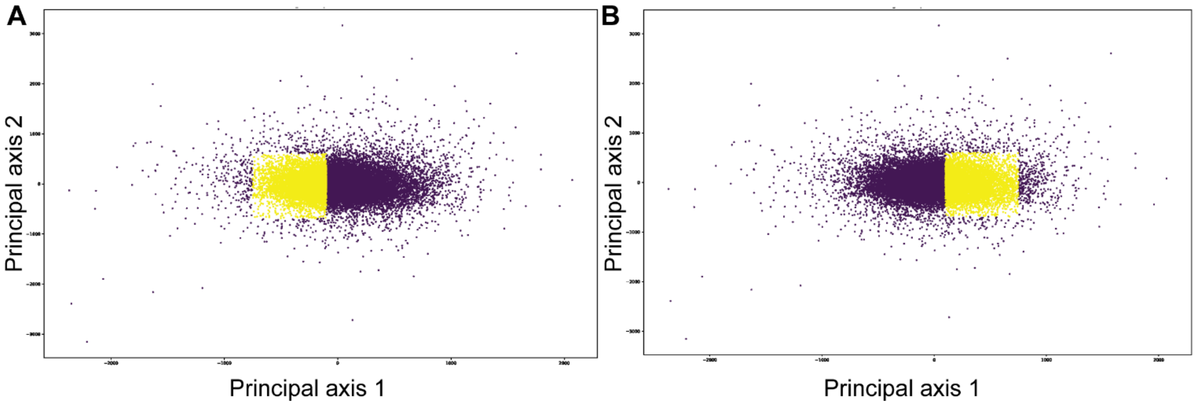

Supplementary Figure S4 Two-dimensional conformational space for the EMPIAR-10016 dataset (cryo-EM single particle images of yeast 80S ribosome-tRNA complexes) obtained by principal component analysis of normal-mode amplitudes inferred from 12,095 images, with panels A and B showing two selected groups of images (yellow) used for the 3D reconstructions shown in **Figure 8A** (4,741 images) and **Figure 8B** (4,219 images), respectively. The groups of images were selected automatically using logical operators on the coordinates of the two principal axes (principal axis 1: [-900, -100] in A and [100, 900] in B; principal axis 2: [-900, 900] in A and B).

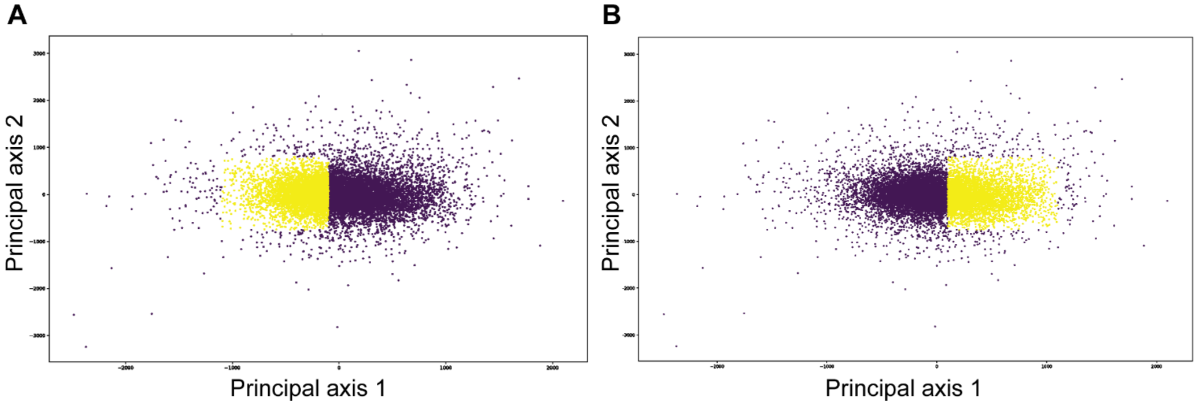

Supplementary Figure S5 Two-dimensional conformational space for the EMPIAR-10016 dataset (cryo-EM single particle images of yeast 80S ribosome-tRNA complexes) obtained by principal component analysis of a combination of normal-mode amplitudes inferred from 12,095 images and HEMNMA-estimated from 10,000 images (the total of 22,095 images represented in this space), with panels A and B showing two selected groups of images (yellow) used for the 3D reconstructions shown in **Figure 8E** (7,870 images) and **Figure 8F** (6,682 images), respectively. The groups of images were selected automatically using logical operators on the coordinates of the two principal axes (principal axis 1: [-1100, -100] in A and [100, 1100] in B; principal axis 2: [-900, 900] in A and B).

SI. Training and validation loss curves

Supplementary Figure S6 shows the training and validation loss curves for the synthetic data experiment shown in the main text.

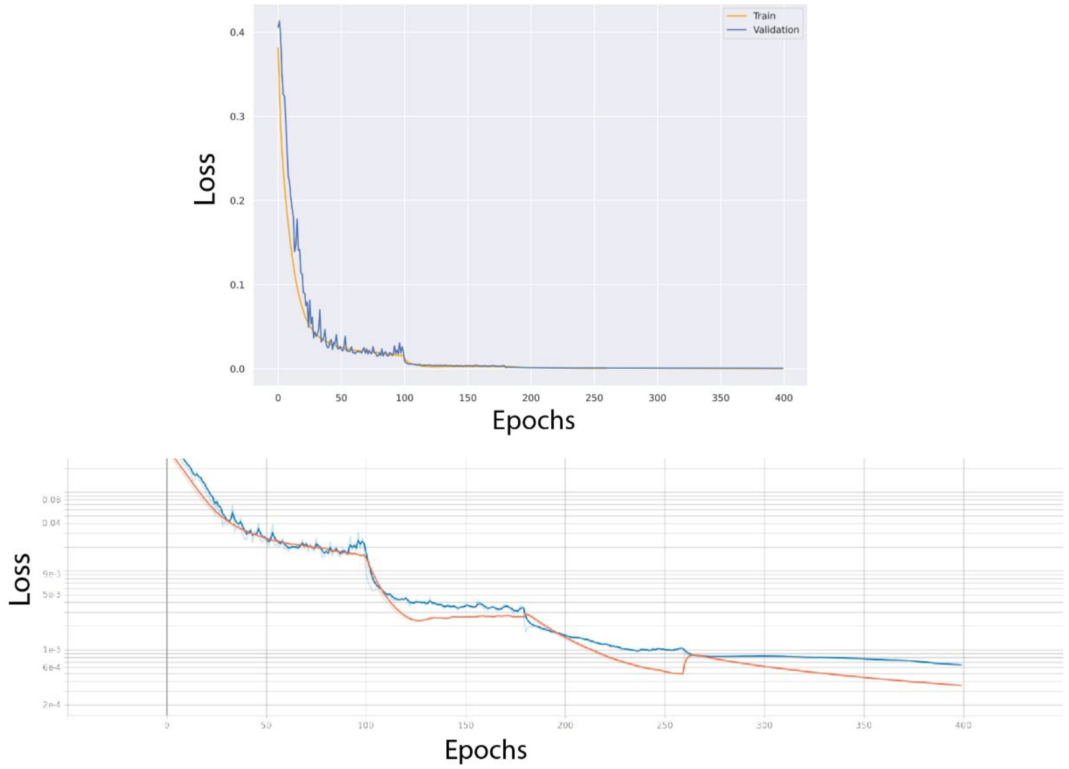

Supplementary Figure S6 Training (orange) and validation (blue) loss curves for the synthetic data experiment shown in the main text. Top: the entire curves. Bottom: loss below 0.1.
